# Bee-Derived Products: Chemical Composition and Applications in Skin Tissue Engineering

**DOI:** 10.3390/pharmaceutics14040750

**Published:** 2022-03-30

**Authors:** Corina Dana Dumitru, Ionela Andreea Neacsu, Alexandru Mihai Grumezescu, Ecaterina Andronescu

**Affiliations:** 1 Department of Science and Engineering of Oxide Materials and Nanomaterials, Faculty of Applied Chemistry and Materials Science, University Politehnica of Bucharest, 011061 Bucharest, Romania; corina.dumitru95@yahoo.com (C.D.D.); grumezescu@yahoo.com (A.M.G.); ecaterina.andronescu@upb.ro (E.A.); 2 Academy of Romanian Scientists, 3 Ilfov Street, 050044 Bucharest, Romania; 3 National Research Center for Micro and Nanomaterials, University Politehnica of Bucharest, 060042 Bucharest, Romania; 4 Research Institute of the University of Bucharest—ICUB, University of Bucharest, 050657 Bucharest, Romania

**Keywords:** wound healing, skin, honey, propolis, royal jelly, beeswax, bee venom, dressings

## Abstract

Skin tissue regeneration is one of the population’s most common problems, and the complications that may appear in the healing process can have detrimental consequences. An alternative to conventional treatments could be represented by sustainable materials based on natural products, such as honey and its derivates (propolis, royal jelly, bee pollen, beeswax, and bee venom). They exhibit significant inhibitory activities against bacteria and have great potential in dermal tissue regeneration. Research in the pharmaceutical field demonstrates that conventional medication combined with bee products can deliver better results. The advantages include minimizing side effects and maintaining the same effectiveness by using low concentrations of antibiotic, anti-inflammatory, or chemotherapy drugs. Several studies suggested that bee products can replace the antimicrobial activity and efficiency of antibiotics, but further investigation is needed to establish a topical mixture’s potential, including honey, royal jelly, and propolis. Bee products seem to complete each other’s deficiencies, and their mixture may have a better impact on the wound healing process. The topic addressed in this paper highlights the usefulness of honey, propolis, royal jelly, bee pollen, beeswax, and bee venom in the re-epithelization process and against most common bacterial infections.

## 1. Introduction

Normally, skin wounds can occur by exposure to fire, hot solids, hot liquids or gases, strong bases/acids, radiation, electricity, or abrasion, puncture, incision, blunt force trauma, and so on. The subsidiary therapeutic aim, in this case, is to prevent and treat the infection, along with ensuring a good recovery by maintaining the right functions [1,2]. Wound healing is a complex, biological process that involves replacing harmed tissue with living tissue. The process of skin healing includes four phases that can overlap: the homeostasis phase, the inflammatory phase, the proliferation phase, and the remodeling phase [3].

Burns are one of the most complex skin wounds and are classified into four degrees, depending on how deeply and severely they penetrate the skin’s surface. First-degree burns involve redness of the epidermis, pain at the injury site, and dryness. Second-degree burns can affect the papillary layer (the upper dermis) or reticular layer (the deeper dermis), presenting a moist appearance with white or yellow coloration and blistering skin. Third-degree burns are described as complete thickness destruction of the epidermis and the dermis and stiff or leathery consistency. After recuperation, the contractures and the scarring will expand. Fourth-degree burns involve severe damage to underlying tissues, ligaments, muscles, tendons, and even bone [4,5].

In recent years, numerous commercially accessible wound dressings have been proposed, but they have some vital barriers consisting of antimicrobial incorporation agents that can have a toxic impact, for example, on a long period of treatment, so the wound recuperation can take longer [6]. So far, many types of wound dressings, including cotton gauze dressings [7], human amniotic membrane [8,9], polysaccharide-based dressings [10,11], and nanofibrous dressings [12,13], have been investigated to assist and enhance the cutaneous wound healing process, ensuring optimum conditions for the restoration of the damaged tissue [14,15]. A commercial dressing doesn’t allow moisture to adhere to the affected surface, the lack of which can harm the epithelium recently formed. Therefore, it has been suggested to use biopolymers (such as collagen [16], chitosan [17], carboxymethylcellulose [18], and alginate [19]) as an alternative to conventional wound dressings, considering that an ideal wound dressing should have the following characteristics: be nonallergenic and nontoxic, preserve the moist environment, promote effective oxygen exchange, protect the wound against microbial organisms, and absorb wound exudates [20]. Biopolymers are biocompatible and swell through liquid uptake by their polymeric networks. In addition, they provide apposite matrixes to promote the healing cascade by mimicking in milieu a moist medium [21].

**Figure 1:**
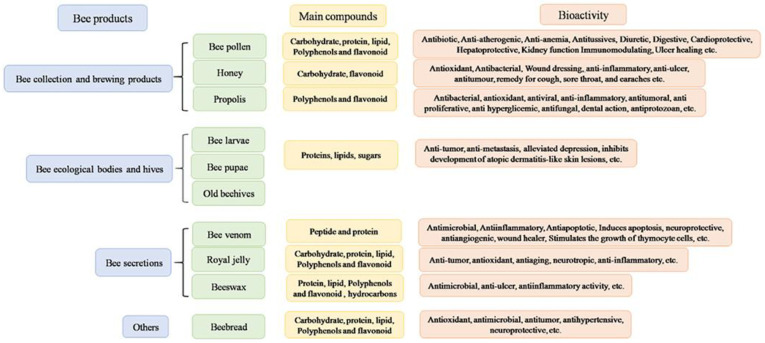
Bee-derived products—main components and bioactivity. Reprinted from [23].

For decades, honeybee products have been used in medical applications, especially in treating primary and secondary wound burns. The most investigated bee products include honey, propolis, bee pollen, royal jelly, beeswax, and bee venom [22]. These bee-derived products demonstrated antibacterial, anti-inflammatory, antioxidant, antifungal, and antiviral properties, which recommended them in such applications (Figure 1). This review aims to present the usefulness of honeybee products in wound healing, correlating their chemical composition with the most important biological properties, and strengthen the existing knowledge regarding the inhibitory activity of these natural products against the most common bacterial infections.

## 2. Bee-Derived Products: Chemical Composition and Biological Properties

Since bee-derived products have a complex chemical composition and low concentration levels of some polyphenolic compounds, it is mandatory to perform several steps prior to the characterization of phenolic compounds and flavonoids. The most common steps to obtain a homogenous and pure sample to analyze are liquid–liquid extraction (aqueous, ethanolic, or in organic solvents) or extraction assisted by microwave and ultrasounds, solvent elimination (e.g., through lyophilization, vacuum distillation, membrane concentration processes, and evaporation), purification, etc. [24,25]. After sample preparation, which is the most time-consuming and susceptible to error step, many techniques for the determination of the complete phenolic profile of bee-derived products were reported. Among the most efficient qualitative analysis methods are high-performance liquid chromatography (HPLC) and capillary electrophoresis (CE), usually coupled with diode-array detection (DAD) and mass spectrometry (MS). MS alone is considered to provide only an unambiguous structure; therefore, it became mandatory to combine it with ultraviolet spectroscopy (UV) and nuclear magnetic resonance spectroscopy (NMR) for the identification of flavonoids [26]. Electrochemical detection and fluorescence detection have also been used in some cases in the analysis of flavonoids due to their sensitivity and selectivity [27,28]. Recently, the profile of phenolic compounds from bee bread, bee pollen, beeswax, and multiflorous honey was determined by ultra-performance liquid chromatography (UPLC) coupled with photodiode array detection (PDA) and electrospray ionization (ESI) tandem mass spectrometry (MS) method [29].

### 2.1. Honey

Honey is a natural substance delivered by numerous honeybee species from all over the world [30]. Based on the diversity of floral sources, about 320 different types of honey were identified. Different types of honey are similar in terms of temperature, rainfall, and seasonal and climatic modifications [31]. Researchers point out that honey is very efficient in major cases of infection and the wound healing management of a burn injury. Therefore, many studies focused on discovering the composition of honey and showing the physical and chemical characteristics that make it useful against a variety of microorganisms [32].

**Figure 2:**
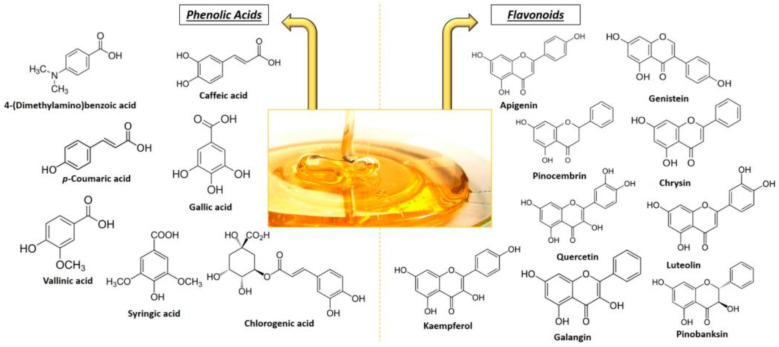
Most common compounds identified in honey. Reprinted from [34].

The chemical composition of honey has received a great deal of attention, and more than 200 compounds were identified in this natural substance. The dry matter of honey’s major constituent is sugar in 90–95%, followed by other constituents such as water, organic acids, and mineral compounds (Figure 2) [33]. In honey’s composition exist numerous important biological bioactive elements such as vitamin A (Retinol), vitamin B1 (Thiamine), vitamin B2 (Riboflavin), vitamin B6, vitamin E (Tocopherol), vitamin K (antihemorrhagic vitamin), Niacin, flavonoids, fatty acids, vitamin C (ascorbic acid), cinnamic acid, pantothenic acid and phenolics, hydroxybenzoic acid, octadecanoic acid, and ethyl ester. Other examples of honey components also include abscisic acid, apigenin, ferulic acid, pinocembrin, and acacetin. Additionally, we can also identify different types of amino acids with physiological importance, such as arginine, proline, cysteine, glutamic, and aspartic acid [31].

In smaller quantities, the presence of phenolic acids and flavonoids were found, which are responsible especially for the unique flavor, appearance, and bioactivities of honey. Phenolic compounds can offer complementary and overlapping modes of action via different types of activities such as antioxidant, antibacterial, and antiviral activities, stimulating the immune system, modulating detoxification enzymes and cholesterol synthesis, reducing platelet aggregation, and also blood pressure. Therefore, their presence in the composition ensures important health benefits of honey. Several investigations have focused on honey’s phenolic profiles and reported a specific correlation between the phenolic content and the antioxidant activity of honey [35]. The phenolic acids that are usually found in honey include p-hydroxybenzoic, gallic, p-coumaric, cinnamic, ferulic, and caffeic acids, and are predominantly found in heather and buckwheat honey. Regarding the flavonoids identified in honey, the most important are chrysin, quercetin, luteolin, myricetin, apigenin, pinocembrin, and pinobanksin (Figure 2) [36].

**Figure 3:**
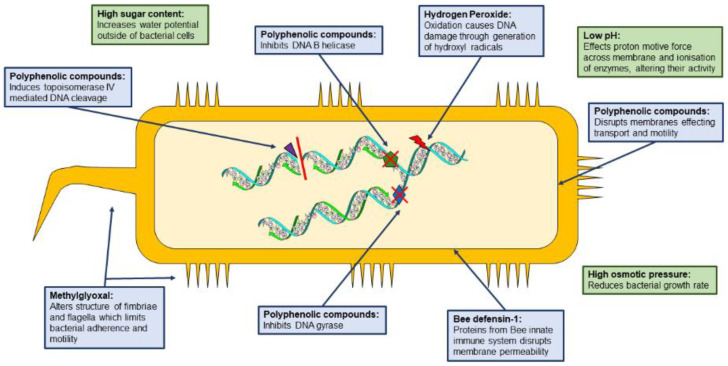
The constituents generating honey’s antimicrobial activity and their mechanisms. Reprinted from [38].

Due to its complex chemical composition, honey has been used in medicinal applications for a long period. The beneficial outcomes of honey represent a good alternative for the management of different wounds. It also contributes substantially to wound healing processes due to its antimicrobial, antioxidant, and anti-inflammatory activities, with a huge impact on the immune system, debridement action, and stimulating function in wound regeneration [37]. These properties are all a result of the synergy between different factors (described in Figure 3), including acidity, high osmotic pressure, presence of phenolic acids, lysozymes, flavonoids, polyphenols, and methylglyoxal. The pH of honey has been documented in a range of 6–7 and appears to offer extra oxygenation to tissues because it can enhance offloading of oxygen from hemoglobin inside the capillaries. This acidification of wounds is responsible for promoting recuperation. Edema and exudates are reduced by the anti-inflammatory action of honey, which can also reduce the pain that occurs by pressing on nerve endings and decreasing the quantity of prostaglandin, resulting in an inflammatory process. Osmolality is also related to honey activity, and its high sugar concentration forms a high osmotic pressure which prevents bacterial growth and proliferation [38].

The antibacterial properties of honey are generally related to the following two main mechanisms: (i) inhibition of the microbial growth by hydrogen peroxide (H_2_O_2_), which is formed by enzymatic activity (for example, glucose oxidase); (ii) inhibition of microbial growth via nonperoxide activities [39]. These nonperoxide activities are based on the action of complex phenols and organic acids, which are well known as flavonoids. The presence of nonperoxide antibacterial factors in honey is proven by the persistent activity in honey products modified with catalase to eliminate the hydrogen peroxide. These antibacterial activities are also influenced by the floral source collected by honeybees, and therefore, not all honey products possess this type of property. Its antioxidant elements are very important and are responsible for the elimination of bacterial infections. Moreover, honey contributes to the production of antibodies and cellular elements implicated in the immunity system [40].

There are some essential benefits offered by honey in the treatment of skin wounds. First, it assures a moist environment because it has the property of being nonadherent. Then, the integrity of the skin surface is maintained and is responsible for providing a barrier for the bacteria, which can prevent cross-infection and contamination with microbes. Numerous investigations indicated that its antibacterial activity eradicates a significant variety of microbes which speeds up the process of wound healing [41].

Another important component of honey is its anti-inflammatory property which includes the debridement of the wound, preventing of scarring, and enhancing recovery of the tissue. Investigations made on affected tissues treated with honey indicated that this bee product is responsible for reducing the quantity of wound exudate. This can also be considered due to the anti-inflammatory activities of honey. Researchers point out that honey can inhibit enzymes that are found in inflammation, such as cyclooxygenase-1 and cyclooxygenase-2. Due to the diversity of its composition, honey is responsible for stimulating or inhibiting the release of specific cytokines, including interleukin-1β, interleukin-6, and tumor necrosis factor-α from human monocytes and macrophages, which are related to wound characteristics. Moreover, honey can decrease or activate the production of reactive oxygen species from neutrophils, which also relies on wound conditions. Additionally, the amount of potent inflammatory constituents such as prostaglandins, including PGE2 (prostaglandin E2), PGF2a (prostaglandin F2a), and thromboxane B2 in plasma, was also reported to decrease in honey-treated wounds [42]. Free radicals’ high content during the inflammatory phase of wound recovery can be extremely harmful and can affect proteins, lipids, and nucleic acids, which are fundamental constituents for the useful activities of all cells. Honey can decrease the damage caused by the free radicals and, in this way, can prevent other tissue necrosis. The activity of the fibroblasts is stimulated by the reactive oxygen species created in the inflammatory phase. Fibroblasts are responsible for collagen fiber production in scar tissue, and prolonging the inflammatory phase can lead to fibrosis and hyper-granulation. The reduction in the inflammatory phase determined by honey minimizes or even prevents hypertrophic scarring [43].

The antioxidant activity of natural honey is determined by the existence of a range of compounds, including flavonoids (quercetin, apigenin, galangin, pinocembrin, chrysin, hesperidin, and kaempferol), phenolic acids (such as caffeic, p-coumarin, ellagic, and ferulic acids), ascorbic acid, catalase, reduced glutathione, tocopherols, superoxide dismutase, maillard reaction products, amino acids, peptides, and selenium [44]. The relative positions of OH groups in the aromatic ring of phenolic acids can determine the antioxidant effect. The quantity and type of these antioxidant elements are typically influenced by the floral source or variety of the honey. Usually, darker honey has demonstrated higher content of antioxidants when compared to a lighter one. The different mechanisms of action regarding antioxidant substances include: inhibiting the enzymes responsible for producing superoxide anions, a decrease of the adverse consequences of reactive oxygen and nitrogen species, radical chain reaction breaking, metal chelation, and also inhibiting the reactive oxidants from being formed [45]. Several investigations point out that antioxidant activities are related to total phenolic concentration. This was suggested for four types of Romanian honey, seven single-origin Italian honeys, and seven types of Slovenian honey [46]. Studies on Portuguese honey have highlighted that the phenolic content of honey, although responsible for its antioxidant activities, also has an important role in determining the antimicrobial effects [47]. Antioxidant properties have also been correlated with the action of several acids, such as citric, gluconic, and malic acids, which determine some processes like chelating metal ions and enhancing the outcome of the antioxidant activity of flavonoids. Intensive research indicated that honey accelerates the wound healing process via antioxidant response by activating AMPK (5′adenosine monophosphate-activated protein kinase) and antioxidant enzymes that can reduce oxidative stress [48].

### 2.2. Propolis

Propolis is a product of resinous substances, with a gummy and balsamic texture, collected by bees from flowers, buds, and exudates from plants. The word propolis is derived from a Greek term in which pro represents “at the entrance to” and polis “city”, indicating that this natural product is used in defense of the hive. Due to its waxy nature and mechanical properties, honeybees use propolis as a binder to keep moisture and temperature stable in the hive all year round and close cracks or open spaces. At high temperatures, propolis is soft, foldable, and very sticky; however, when it is cold, and especially when it is frozen or almost frozen, it becomes hard and fragile [49].

**Figure 4:**
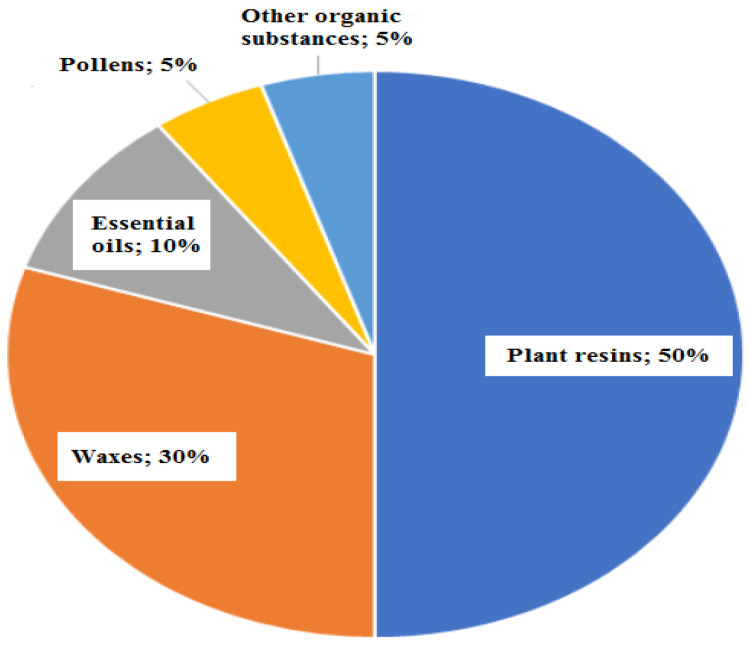
Compositions of propolis. Reprinted from [52].

The color of propolis is often dark brown. According to the resin sources found in the areas near the hive, it can also have other colors like green, red, black, and white. For example, in northern temperate climates, bees collect resins from trees like poplars and conifers. The most widespread propolis types are green, poplar, birch, red, Mediterranean, Clusia, Pacific, Tunisian, Iranian, and Egyptian propolis, all different in geographic origin and plant source (Table 1). Typical propolis contains resin and balsam, wax, essential and aromatic oils, pollen, and other organic substances (Figure 4) [50].

**Table 1:** Most important propolis types (resumed from [51]).

Propolis Type	Origin	Major Constituents
Poplar	Europe, North America, and the nontropical regions of Asia	Flavonoids and phenolic acid esters (flavones, quercetin derivates, pinocembrin derivates, and daidzein)
Red propolis	Cuba, Mexico, Brazil	Derivatives of *p*-coumaric acids, artepillin C, different caffeoylquinic acids, and lower amounts of flavonoids
Pacific	Taiwan, Japan	C-prenyl-flavanones
Mediterranean propolis	Greece, Malta, Crete, southern Italy	Diterpenes

The chemical composition of propolis can vary depending on the production region, the availability of the sources of collection of plant resins, the genetic variability of the queen bee, the technique used for production, and the season in which the propolis is produced. The studies on the chemical composition of propolis from Europe have identified only flavonoids and phenolic compounds. It was found that propolis from temperate climatic zones, like Europe, nontropical regions of Asia, and North America, is obtained mainly from the bud exudates of Populus species and their hybrids and has in composition flavonoids, phenolic acids, and their esters. Instead, the propolis from tropical regions is rich in prenylated benzophenones, diterpenes, and flavonoids due to the absence of poplars and birches [53,54,55].

The propolis specific to the temperate zone is named poplar propolis because it is mainly obtained from the exudates of Populus buds. Birch propolis originates especially from Russia and is not similar to poplar propolis. Pacific propolis is a type of propolis originating from Taiwan, Japan, and the Solomon Islands [49]. Brazilian green propolis is extracted from a Brazilian plant named *Baccharis dracunculifolia DC* (*Asteraceae*) and represents the most studied type of propolis. Brazilian green propolis is composed of prelene, phenylpropanoids such as artpillin C, and diterpenes. The dominant constituents of Brazilian green propolis include caffeoylquinic and prenylated cinnamic acids such as artpillin C and baccharin, which possess antioxidant and inhibitory characteristics against some enzymes. It has been shown that artpillin C can inhibit neutrophil mobilization in the abdominal cavity [50].

Red propolis is similar to green propolis and comes from Apis mellifera, but the composition is different because the bees collect another plant (*Dalbergia ecastophyllum*) to obtain it. The main chemical components of red propolis are phenylpropanoids, terpenes, flavonoids, aromatic acids, and fatty acids. In addition, inorganic elements such as iron, copper, aluminum, manganese, calcium, vanadium, and silicon are also found in this type of propolis. Flavonoid content is higher in red propolis than in green propolis [56].

In propolis, around 300 compounds are present such as phenolic acids, cinnamic acid, caffeic acid, terpenes, flavonoids, esters, amino acids, sugar, sterols, steroid hydrocarbons, minerals, aliphatic hydrocarbons, sesquiterpene, and triterpene hydrocarbons (Table 2). Propolis is a lipophilic substance, and it has a distinct odor; its oil shows adhesive properties and has a strong reaction with skin proteins [57]. Terpenoids represent 10% of the composition and are responsible for the odor, as they are volatile components of plants and are related to the biological properties of propolis. Terpenoids include diterpenes such as ferruginol, junicedric acid and its derivatives, pimaric acid, monoterpenes including terpineol and camphor, and triterpenes such as amyrone and the derivatives of lupeol and lanosterol [49].

As previously mentioned, propolis is partially soluble in water and cannot be used as a raw material. Therefore, to use it in various applications, it must be purified through extraction with solvents to eliminate the inert material and maintain the polyphenolic fraction. Flavonoids and phenolic acids are considered more efficient in the healing process when compared with the effects of the other propolis components. Propolis extracts are generally obtained via typical methods, such as ethanolic or aqueous extraction or Soxhlet [59,60].

Regardless of the plant source (plant species and geographical origin) and the chemical or biological compositions, propolis activity, especially antimicrobial activity, has always been identified. Therefore, propolis plays an important role in the hive: it represents a “chemical weapon” against pathogens and microorganisms, a constant threat to the vital health status of this busy “city”, vulnerable to the invasion of a series of enemies and diseases proliferation. Despite this important function and due to the diversity of plants, there are some types of propolis that contain many chemical constituents related to antimicrobial properties but also other valuable bioactivities. The biological properties include antibacterial, anti-inflammatory, antimycotic, antioxidant, antiviral, antiprotozoal, local anesthetic, cytostatic, immunostimulatory, and hepatoprotective features. The active propolis factors responsible for its biological properties are well defined and vary with the sample of propolis, dose, and the solvents used for extraction. Flavonoids and esters of phenolic acids are considered bioactive compounds, and the ratio of the combined reagents in propolis is important for its effect.

**Figure 5:**
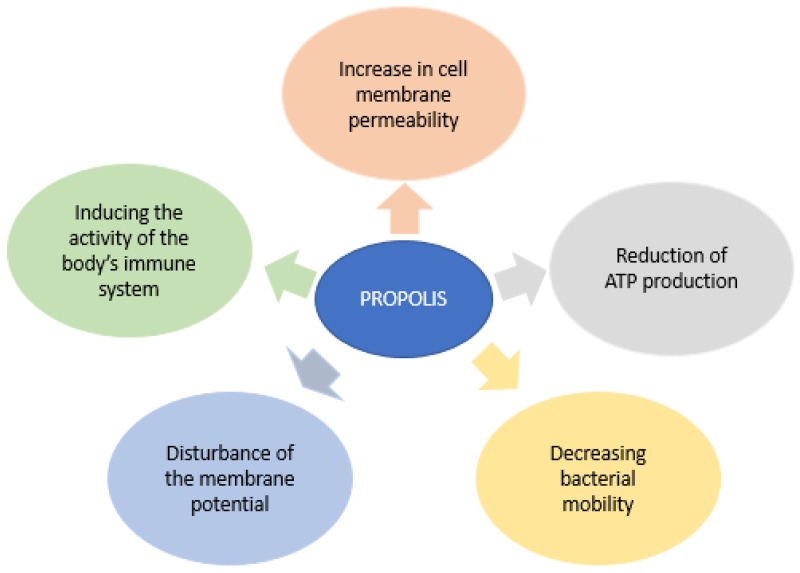
Antibacterial activity of propolis. Reprinted from [52].

Propolis, which is well tolerated with rare allergy incidents and no toxicity, is mentioned as an excellent candidate for managing wounds, for the growth of skin cell proliferation, activation, and growth capacity. Several studies confirm the therapeutic efficacy of propolis through quantitative and qualitative analyzes of type I and III collagen, indicating that propolis could have environmentally friendly biochemical effects in support of re-epithelization. Recent findings have demonstrated that oxygen is required for wound disinfection and for wounds to heal, but also oxygen-dependent redox-sensitive signaling processes play an essential role during the healing process. The interactions between free radicals in the skin and the vicinity of tissues may also be responsible for some toxic effects and changes in their structure. Other studies show that propolis accelerates the repair of affected tissue by stimulating the wound bed by remodeling the matrix, and the changes observed in the content of the extracellular matrix after the application of propolis may be related to the ability of its flavonoid compounds to reduce lipid peroxidation and prevent cell necrosis. The biological activities of propolis involved in wound healing and tissue regeneration could be correlated with its antimicrobial properties as well as its anti-inflammatory and immunomodulatory properties (Figure 5) [61].

Propolis is regarded as more effective against Gram-positive bacteria than Gram-negative bacteria. Gram-negative external membrane is determined by the species and indicates the porin or lipopolysaccharide amount of the external layer; in addition, the constituents of propolis could be damaged in the bacterial suspension with the aid of hydrolytic enzymes released by the bacteria. Propolis components may enhance membrane permeability and repress bacterial motility, and these mechanisms can be correlated with the antimicrobial activity of propolis. Propolis may influence ion permeability of the internal bacterial membrane and cause the dissemination of the membrane potential. The electrochemical gradient of protons throughout the membrane is vital for bacteria to preserve adenosine triphosphate synthesis, membrane transport, and motility to stay viable. Propolis possesses effects specifically on microorganisms in vitro but can also activate the mechanisms involved in the microorganisms’ damage and stimulate the immune system in vivo [62].

The ways in which propolis works are determined by the interplay between phenolic and different constituents, including pinobanksin, pinocembrin, and galangin. Moreover, the antibacterial activity is attributed to its active compounds, such as aromatic compounds, especially caffeic acid and flavonoids. In addition, propolis has a bactericidal role to stop the division of the bacterial cell, damage the cell wall bacterial cytoplasm, and stop protein synthesis [63]. Pinocembrin is the component of propolis that shows antibacterial activity against *Streptococcus* spp., while artepillin C, p-Coumaric acid, and 3-phenyl-4-diydrocinnamylocinnamic acid strongly limit bacterial glycosyltransferase against *Apigenin* and *Helicobacter pylori* [61].

Flavonoids can determine the antibacterial activity of propolis and can limit the resistance of bacterial cells to different antibacterial agents by expanding the permeability of the internal bacterial membrane and disseminating its potential. These discoveries encourage a synergistic impact that takes place between propolis and different antibiotics. It was demonstrated that propolis action can lead to fractional bacterial lysis and inhibit protein synthesis [64].

**Figure 6:**
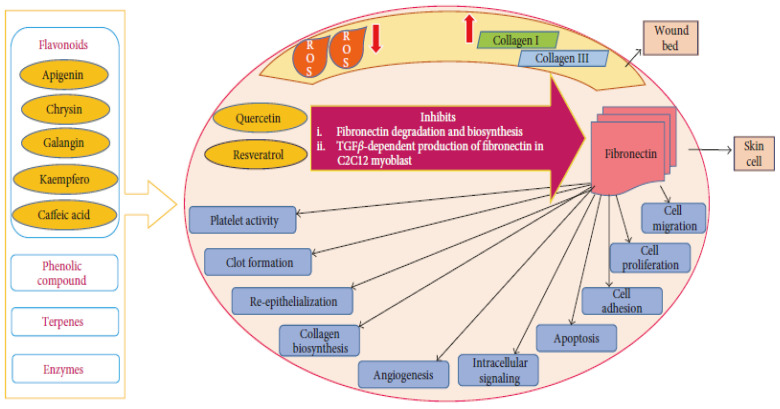
Molecular mechanism targeting the wound healing activity of propolis. Reprinted from [69].

The antioxidant agents from propolis can be correlated with its immunomodulatory activities. An example of powerful antioxidants present in the composition of propolis is flavonoids. Various antioxidants and enzymes play an essential role in adjusting the generation rate and cleaning the oxidants. Severe skin wounds are related to the release of inflammatory mediators and reactive oxygen/nitrogen species. The formation of free radicals in the skin can specifically influence the characteristics of an element of the cell membrane or intracellular organ or determine an inflammatory signaling cascade followed by the generation of various mediators of cell damage. In this manner, the utilization of antioxidant agents has indicated a positive effect on healing [65,66,67]. The mechanism of the antioxidant property of propolis is attributed to phenolic constituents that can donate hydrogen ions to free radicals to protect the cell from oxidation reactions. Propolis can eliminate free radicals, which are the main cause of lipids, nucleic acids, and proteins oxidation (Figure 6). Vanillin and phenolic acids are other constituents of propolis that can enter the epidermis or the dermis and protect them against free radicals, resulting after radiation or before the maturation of dermal cells aging.

Propolis accelerates skin wound healing by stimulating epithelial recovery, modulating extracellular matrix deposition, and facilitating granulation tissue formation. Subsequently, the antioxidant properties of propolis may be responsible for its defensive effects in cutaneous diseases. Burn wounds treated with propolis were found to have lower concentrations of free radicals [68].

The antioxidant properties of phenolic acids and flavonoids are supplemented by their anti-inflammatory activity. Inflammation can be described as an interplay between the immune system and injured tissues planned to repair homeostasis through complex signaling pathways. The major constituents responsible for the anti-inflammatory activity of propolis are represented by flavonoids, steroids, amino acids (CAPE), terpenoids, phenolic acids, and their esters. The most important mechanisms related to the anti-inflammatory activity of propolis include the following actions: the inhibition of cyclooxygenase and consequent inhibition of prostaglandin biosynthesis; free radical scavenging; inhibition of nitric oxide synthesis; reduction in the concentration of inflammatory cytokines; and immunosuppressive activity [70]. The anti-inflammatory activity controls nicotinamide adenine dinucleotide phosphate (NADPH) oxidase, ornithine decarboxylase, myeloperoxidase activity, hyaluronidase from guinea pig mast cells, and tyrosine-protein kinase. The outcomes of these activities are reflected in the following abilities limiting leukotriene and prostaglandin generation by white blood cells and retarding myeloperoxidase activity, ornithine decarboxylase, tyrosine-protein-kinase, and NADPH oxidase [63]. Propolis’s polyphenols and flavonoids constituents can remove free radicals from tissues. CAPE is the most investigated active biological constituent of propolis, responsible for decreasing inflammation by repressing the generation of chemokines and cytokines [71]. Burning is described as a post-traumatic inflammatory process that can lead to tissue injuries, infections, generation of toxic inflammatory mediators, oxidants and proteases, wounds, and even mortality. Because CAPE can reduce plasma levels of malondialdehyde and xanthine oxidase activity in patients affected by burns, it is regarded as a helpful agent in treating burn wounds.

### 2.3. Royal Jelly

Royal jelly (RJ) can be described as a white and viscous substance, soluble in water, having a density of 1.1 g/mL, similar to jelly, which is a type of hypopharyngeal and mandibular gland secretion from the worker bees. It is also called the “superfood” that is exclusively consumed by the queen bee. After hatching, royal jelly is also fed to the honeybee larvae and helps feed the young bees. Alongside the fact that it is typically the nutriment for the queen bee throughout their entire life cycle, it is also the specific food consumed by the immature young larvae in their first 2–3 days of maturation [69]. The morphological change of a larva into the queen bee is possible due to the presence of the royalactin compound of royal jelly. Due to this superfood, the queen bee lives longer than the worker bees. Royal jelly is extensively used as a dietary nutritional complex against a wide range of chronic health conditions. Moreover, in modern and traditional medicine, royal jelly is regarded as an efficient treatment for human beings [72,73]. Forager bees collect pollen with their hind leg corbiculae, and the following step adds nectar to obtain pollen pellets. The pollen pellets are deposited and packed by the worker bees into cells surrounding the brood area, and therefore, bee bread is obtained. Nurse bees possess enlarged food glands and produce RJ after they feed themselves with honey and bee bread [74].

RJ’s chemical composition is influenced by seasons and medium characteristics around the region where the bees inhabit and forage. It also depends on the race and caste of the honeybee, on the time when the royal jelly is collected, and on physiological and metabolic variations between the nurse bees [75]. RJ is an acid colloid (3.6–4.2 pH), and the main constituents include water, proteins, sugar, lipids, vitamins, and different mineral salts. RJ contains water in a range of 60% to 70%, carbohydrates from 11% to 23%, proteins from 9% to 18%, lipids from 4% to 8%, and reduced quantities of vitamins and mineral salts [76]. From a chemical point of view, royal jelly is an emulsion of sugars, proteins, and lipids in water. It contains about 1.5% mineral salts, mainly iron, copper, zinc, calcium, manganese, potassium, sodium salts, and also small quantities of flavonoids, polyphenols, and vitamins, including inositol, biotin, niacin, folic acid, pantothenic acid, thiamine, riboflavin, and vitamin E. The variety of flavonoids found in royal jelly includes flavanones (hesperetin, naringenin, and isosakuranetin), flavones (such as cacetin, apigenin, and its glucoside, chrysin, and luteolin glucoside), flavonols (kaempferol and isorhamnetin glucosides), and isoflavonoids (genistein, coumestrol, and formononetin) [77,78]. In addition to water, protein, carbohydrates, lipids, mineral salts, and vitamins, which are the most important constituents of RJ, some bioactive elements such as 10-hydroxy-2-decenoic acid (10-HDA), adenosine, acetylcholine, polyphenols, adenosine monophosphate (AMP) N1 oxide, and some hormones enter in the composition RJ. This type of bee product has a significant role in various biological and health-promoting activities [79].

**Figure 7:**
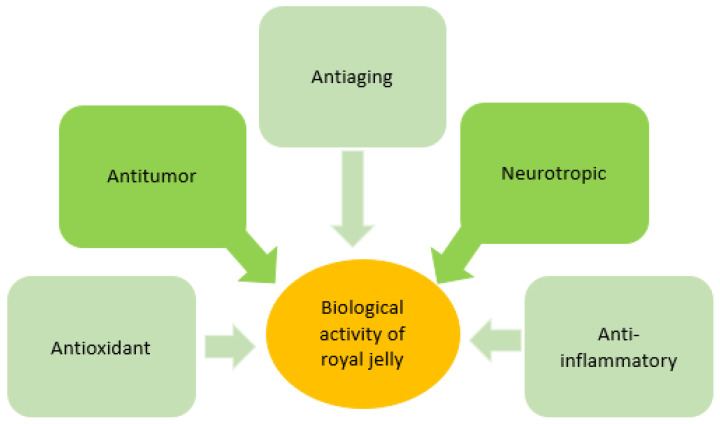
Biological properties of royal jelly (adapted from [69]).

RJ is considered to possess different pharmacological activities in vitro, in vivo in experimental animals, and in clinical studies, including antitumor, antimicrobial, antihypercholesterolemic, and anti-inflammatory activities, vasodilative and hypotensive activities, and an increase in growth rate (Figure 7).

The clinical significance of RJ has been documented in ancient times, and it was demonstrated that an aqueous solution of pure RJ possesses a strong antibacterial activity towards a wide range of bacteria. Several studies point out that the agent responsible for the antibacterial property of RJ is a special molecule of 10-hydroxy-2-decenoic acid (10HDA), which is the essential fatty acid found in RJ [80]. The carboxylic acids that are found in RJ are known to apply antimicrobial features against fungi, Gram-positive, and Gram-negative bacteria. 10-HAD possesses strong antibacterial activity, specifically against *Escherichia coli* (*E. coli*), *Bacillus subtilis* (*B. subtilis*), and *Staphylococcus aureus* (*S. aureus*) [81]. Additionally, royalisin, a 51 amino acid peptide, which is like the hemolymph defensin-1, was reported to have antibacterial activity against numerous Gram-positive bacteria, such as *Micrococcus luteus*, *Staphylococcus*, *B. subtilis*, *Streptococcus*, *Sarcina lutea*, *Lactobacillus helveticus*, *Clostridium*, *Paenibacillus larvae*, *Corynebacterium*, and *Leuconostoc*. However, against *Serratia marcescens* and the Gram-negative *E. coli*, no inhibition has been identified. Royalisin also determines the antifungal activity against *Botrytis cinerea* [82]. Moreover, it has also been found that the antimicrobial peptide royalisin can protect wounds against infection.

The anti-inflammatory action of RJ is indicated by decreasing exudation and collagen formation in granulation tissue and increased wound healing in streptozotocin-induced diabetic rats. RJ is also responsible for reducing the recovery period in desquamated skin injuries [80]. The wound healing response is promoted by RJ, which can manage dermal infection caused by methicillin-resistant *S. aureus* (MRSA). Water-soluble proteins of RJ and its fractions initiate transitory and proliferative impacts on a human epidermal keratinocyte in a scratch wound model [83].

The defensin-1 peptide from RJ has a significant role in skin regeneration and cutaneous wound closure by stimulating matrix metalloproteinase-9 secretion and keratinocyte migration [84]. Royal jelly stimulates re-epithelization of the wound. The accelerated production of MMP-9 (matrix metalloproteinase-9) is determined by keratinocytes after applying a water extract of royal jelly. After using a water extract of royal jelly, increased wound closure rates and elevated keratinocyte migration was detected. MMP-9 production is stimulated by defensin-1, which can enhance re-epithelization and wound closure. Defensin-1 has a similar role as in honey. It determines cutaneous wound closure by increasing MMP-9 secretion and keratinocyte production [85]. RJ enhances skin regeneration after wounds, while major royal jelly protein 3 (MRJP3) represses the production of pro-inflammatory cytokines and enacts keratinocytes involved in wound treatment. The inflammation is reduced due to the hormone-like effects of royal jelly, while collagen secretion is determined by 10HDA. RJ has a strong impact on the healing period of desquamated skin injuries by reducing it along with exudation. Moreover, it can accelerate the wound healing period and also enhance the formation of collagen in granulation tissue formation [86].

It is well known that RJ is obtained after the digestion of bee pollen by natural enzymes in the honeybee, and RJ has a similar composition to the bee pollen, including all pollen phenolic compounds. Therefore, phenolic compounds, peptides, and proteins are those that determine the antioxidant activity of RJ [87]. The anti-inflammatory activity of royal jelly makes it effective in several health issues such as periodontal diseases’ inflammation of the oral cavity, throat, and tongue. Its ability to suppress the secretion of pro-inflammatory cytokines determines the anti-inflammatory activity and wound healing [85]. Peptides with amino acid residues possess radical scavenging features. The antioxidant effects of royal jelly make it suitable as an anti-aging product [79].

Antiallergenic and immunomodulatory features of royal jelly are associated with the fatty acid properties isolated from it. 3-10-dihydroxydecanoic acid and 10HDA inflect immune response and decrease the concentration of IL-10 and IL-2. Royal jelly’s anti-inflammatory and immunomodulatory activities are suitable in treating atopic dermatitis, hyperkeratosis, epidermis and dermis inflammation, or hypertrophy, most probably via a combination of TNF-specific low adjustment of IFN-gamma unique secretion and excessive adjustment of nitric-oxide synthase expression. Another element in RJ is 10-hydroxy-trans-2-decenoic acid, which stimulates fibroblast collagen production by inducing the secretion of transforming growth factors. Therefore, royal jelly influences collagen secretion, which represents a significant component that supports the skin. Royal jelly is particularly moisturizing and impacts hydration of the stratum corneum by retaining water in it. As a result, the skin becomes more elastic and highly moisturized [85].

### 2.4. Bee Pollen

Pollen is the male gamete produced by the anthers of seed-bearing plants (gymnosperms and angiosperms). These male gametes—germ cells—fuse with female gametes for fertilization. Most flowering plants require pollinators to transfer from male anther to female stigma, known as pollination. Bees play an important role as a pollinator. Adult workers forager honeybees collect and gather pollen from the field, bring it back to the hive, and use it as a primary food source. Thus, pollen plays a crucial role in maintaining pollinator health [88]. Bee pollen is the main source of important nutrients and phytochemicals that honeybees collect to feed their larvae. It is a mixture of floral pollen, nectar, and mouth secretions of bees, accumulated as pellets of different colors, sizes, and morphology [89].

**Figure 8:**
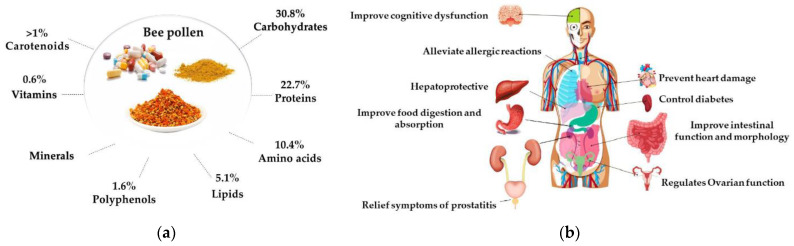
Bee pollen: (**a**) main constituents; (**b**) therapeutic applications (adapted from [92]).

The chemical composition of bee pollen depends on the plants the worker bees gather the pollen from and may vary from hour to hour, day to day, week to week, colony to colony, even in the same apiary. Although there is no specific chemical composition, the average composition is reported to be 40–60% simple sugars (fructose and glucose), 20–60% proteins, 3% minerals, and vitamins, 1–32% fatty acids, and 5% diverse other components including considerable amounts of vitamins, flavonoids, and phenolic acids (Figure 8a) [90]. Due to the presence of different types of metabolites, this bee product has attracted attention in the medical field and has been used in various therapeutic applications. (Figure 8b) [91].

The bee pollen collected by worker bees contains nutrients that are usually needed by the body and bioactive substances such as flavonoids, carotenoids, phospholipids, and polyunsaturated fatty acids. Among them, flavonoids have antibacterial, antiviral, antitumor, antioxidation, anti-inflammatory, analgesic, anti-aging, antiradiation, and other pharmacological activities alongside liver, heart, and bone protection [93]. The physicochemical properties of bee-collected pollen can be affected by processing techniques and storage conditions. Pollen freshly collected by honeybees contains water from 15% to 30% (*w*/*w*). Therefore, it needs to be promptly processed to boost its physicochemical stability, avoiding microbial development [94]. The pollen quality can be roughly judged based on its color linked to plant pigments like carotenoids and anthocyanins, inherently found in different concentrations in pollen. The bee pollen contains all shades of color, from white to black. However, the pollen collected from the same plant source may have different colors, and sometimes, the bee pollen from various botanical origins can be similar [95]. The pollen collected in the spring is significantly different in terms of amino acid content compared to the pollen load collected by bees in the summer. The carotenoids and vitamin C contents in pollen loads collected from various plants are differentiated [96]. In the process of healing burn wounds, the bee pollen ointment helps by preventing infection in the area of newly formed tissue [97].

Bee pollen is often referred to as “nature’s most perfect food” because it is a complete protein (typically containing 10–35% total protein), meaning it contains all eight essential amino acids. It also provides B vitamins, vitamin C, carotenes, minerals, DNA, RNA, numerous flavonoid molecules, and plant hormones. Pollen has similar nutritional qualities to propolis and royal jelly but considerably higher levels of different biologically active compounds, such as phospholipids and vitamins.

Bee pollen possesses around 70% of biologically active substances. This honeybee product is demonstrated to exhibit multiple effects such as antibacterial, cardioprotective, nutritive, hepatoprotective, immunostimulant, antioxidative, anticancerous, antianemic, and anti-inflammatory effects. The most important activities of bee pollen are included in Table 3 [98].

Due to the presence of flavonoids and phenolic acids in bee pollen, the ethanol extracts of this bee product exhibited an important antibacterial activity against Gram-negative and Gram-positive bacteria but also pathogenic fungi. Flavonoids have an inhibitory activity on bacteria by affecting their metabolism. The main mechanism refers to the formation of various complexes at the surface of the bacterial cell wall after exposure to polypeptides and enzymes, which affect the cell wall integrity and block the ion channels and the electron flow implicated in the synthesis of adenosine triphosphate (ATP) [98].

The previous antimicrobial studies of bee pollen exerted strong antimicrobial activities on several bacterial strains: *E. coli*, *Bacillus cereus* (*B. cereus*), *B. subtilis*, *S. aureus*, *Salmonella enteritidis* (*S. enteritidis*), *L. monocytogenes*, and *P. aeruginosa*. Additionally, these extracts also exhibited antifungal effects on various types of bacteria including *Rhodotorula mucilaginosa*, *Aspergillus fumigatus*, *Candida glabrata*, *Aspergillus flavus*, *Candida krusei* (*C. krusei*), *Aspergillus niger*, *Candida tropicalis*, *Candida albicans* (*C. albicans*), *Candida parapsilosis*, and *Geotrichum candidum.* Moreover, researchers found that the lipophilic fractions of bee pollen originating from three different floras could resist the growth of several Gram-positive bacteria. The antimicrobial and antifungal properties of bee pollen help prevent and aid in managing bacterial and fungal infections, respectively [30]. The lowest inhibitory activity of bee pollen was registered against *P. aeruginosa* with various research papers indicating that the minimum inhibitory concentration (MIC) values reported against *S. aureus* were lower than against *P. aeruginosa*. An explication could be related to the structural differences of the cell wall of the Gram-negative bacteria, which is more complex than in the case of Gram-positive bacteria [99].

Bee pollen antioxidant properties depend on the origin of the flora and the presence of phenolic and polyphenolic components, one of the most important being flavonoids. Samples of bee pollen extracted from various flora were investigated, and the most common constituents found were kaempferol, flavonoids tricetin, myricetin, luteolin, isorhamnetin isoquercetin, and selagin. The phenolic composition is similar for these constituents, but a distinctive ratio was observed. Researchers focused more on the scavenging activity and ROS’s antioxidant power. Both of these bioactivities are having an impact on the potentiation of clinical studies on various diseases, including cardiovascular problems, diabetes, obesity, hypertension, obesity, or even degenerative pathologies (Alzheimer’s disease, Huntington’s disease, arthritis, and Parkinson’s disease). Studies revealed that an increase in ROS concentration in cells leads to oxidative stress. The increase can be induced by environmental factors, including exogenous and endogenous types. Cell membrane and DNA destruction are a result of increased ROS levels. These effects are related to cellular response and are responsible for chronic inflammation generation [100].

Another important property of bee pollen is its significant anti-inflammatory activity. Its relevance is similar to anti-inflammatory medicines, including indomethacin, naproxen, phenylbutazone, and analgin. The anti-inflammatory activity has a complex mechanism that includes inhibition activity of lipoxygenase and cyclooxygenase. These enzymes can transform arachidonic acid into prostaglandin and leukotrienes, which have a toxic effect by creating acute and chronic inflammatory conditions in tissues.

Substantial evidence suggests that pollen compounds (polyphenols or flavonoids) may exert beneficial effects on numerous cells (macrophages, T cells, B cells, NK cells, hepatocytes, mast cells, basophils, neutrophils, and eosinophils), which play a crucial role in host defense against invading pathogens and in inflammatory processes. The anti-inflammatory action of flavonoids may result from the activity of quercetin, which is known to inhibit arachidonic acid metabolism. A decrease in the arachidonic acid level reduces the level of pro-inflammatory prostaglandins and provides an anti-inflammatory effect. As a result, good effects for local pain elimination and prevention of platelet aggregation are observed after the application of bee pollen [101].

### 2.5. Beeswax

Beeswax can be defined as a complex lipid incorporating organic constituents displayed in a liquid form, being a result of the specialized wax glands of the bees. Beeswax is initially glass-clear and colorless, becoming opaque after mastication and adulteration with pollen by the hive worker bees. In addition, the wax becomes progressively more yellow or brown with the incorporation of pollen oils and propolis. Beeswax has a relatively low melting point range of 62 °C to 64 °C (144 °F to 147 °F). If beeswax is heated above 85 °C (185 °F), discoloration occurs. The taste of beeswax is normally pleasant and is not specific—any unpleasant taste is a sign of quality deterioration due to foreign matter. The odor should be pleasant and honey-like [102]. In contact with air, the liquid form is transformed into a solid form of scales [103].

Wax is collected and reused in apiculture and other sectors, such as food, chemical, pharmaceutical, or cosmetics. In the agri-food industry, beeswax is used as a food additive (E901), as a glazing agent in the preparation of pastries, for the treatment of some fruits, as a food supplement, and as a flavor carrier. Beeswax plays a role as a binder, thickener, and drug carrier and releases retardant in pharmaceutical preparations [104,105,106]. It is most appreciated in the medical field. Cytokines in the skin cells are increasing due to beeswax’s special properties, such as antibacterial and antioxidant activities [107].

Its chemical composition is represented by a combination of almost 300 constituents with a different ratios, such as fatty acid esters (approximatively 67%), free fatty acids (12–14%), diesters, hydrocarbons (among 12–16%), fatty alcohol (approximatively 1%), and exogenous substances (propolis and pollen residues, and a reduced number of floral constituents) [103,104]. The structure of beeswax is crystalline. The crystallization of beeswax depends on the storage. The crystallization process increases upon storage of wax until 3–4 months, while at the same time, its stiffness and elasticity increase. The mechanical properties of wax are important in connection with its use as “the house of the bees”. Beeswax is also insoluble in water and resistant to many acids. It is soluble in most organic solvents such as acetone, ether, benzene, xylol, toluene, benzene, chloroform, and tetrachloromethane. However, at room temperature, it does not fully dissolve in any of these solvents, but upon heating above the wax melting point, it is readily soluble in all of them and also in ethanol [108].

Beeswax is also a product that has positive effects on the wound healing process when used in wound care and treatment, thanks to flavonoids and antioxidants. When applied topically, the antibacterial and antifungal compounds from its composition can affect the production of cytokines by skin cells [109]. Moreover, beeswax has anti-inflammatory and antimicrobial activities, especially in wound management as well as anti-stress and antioxidant effects [110].

Most of the studies published in recent years focus especially on the antibacterial properties of natural products and mostly on beeswax among all the other bee products. Unprocessed beeswax was observed to have antibacterial inhibition against different types of bacteria and also *C. albicans* yeast. It was demonstrated that the efficiency of beeswax can be detected against some Gram-positive bacteria including *S. epidermis*, *Streptococcus Pyogenes* (*S. pyogenes*), and *S. aureus* but also on *P. aeruginosa*, *B. subtilis*, and *E. coli* as part of Gram-negative bacteria. A smaller impact was shown among *C. albicans* while against *Proteus mirabilis* and *Salmonella typhimurium*, beeswax proved to have no effect. Additional research and testing were conducted especially on the mixture of beeswax with other honeybee products/natural products, to evaluate their antimicrobial efficiency. The synergy between honey, olive oil, and beeswax was demonstrated to have an important impact against *C. albicans* and *S. aureus* [111,112].

Another combination of beeswax with propolis extract, honey, and olive oil was applied to healing chemotherapy-induced mucositis, and the results indicated it to be more efficient, especially in patients with severe degrees of mucositis. The mixture of beeswax with honey and olive oil, in particular, indicated similar activity to Nystatin cream when is applied against *C. albicans*. Therefore, the combination of Nystatin cream with this type of natural mixture could enhance the healing process. This mixture was also demonstrated to have better efficiency in wound management of canine deep second-degree burns with great impact in the veterinary field [111,113].

### 2.6. Bee Venom

Another honeybee product that has been used as a natural product in the treatment of various diseases due to its special properties is bee venom (BV). This hive product is obtained from the venom gland of the honeybee [114]. Bee venom chemical composition was investigated and was found to have among 20 bioactive substances, which are responsible for their specific properties such as antitumoral, antinociceptive, antirheumatic, anti-inflammatory, neuroprotective, antiarthritic, antimicrobial, and antidiabetic. These substances can be classified as peptides, bioactive amines, sugars, phospholipids, enzymes, amino acids, pheromones, and minerals. The most important peptide from its composition is melittin, followed by apamin and adolapin [115]. Melittin represents 40–60% of bee venom composition, the main peptide constituent. This peptide has a hydrophilic carboxyl-terminal region (with lytic activity) and a hydrophobic amino-terminal region (no lytic activity). Due to the amphipathic activity of this peptide, bee venom is soluble in water in both forms: tetrameric and monomeric. This property helps melittin to integrate into membranes by damaging the phospholipid bilayers [116].

Apamin represents the smallest neurotoxin from bee venom composition, being an 18-amino acid peptide. The percentage of this peptide in bee venom dried form is about 37–44% and has a great contribution to the biological function of this bee product. In various studies, it was reflected that apamin possesses important antinociceptive and anti-inflammatory functions. The peptide can block Ca^2+^-activated K^+^ channels, and according to this action, the cell membranes permeability is affected by the potassium ions. Central nervous system function can also be affected due to apamin ability to enter across the blood–brain barrier [117]. PLA2 (phospholipase A2) enzyme is the main allergen from bee venom composition, and its activity is related to pain and inflammation [118].

Researchers have investigated the antimicrobial activity of bee venom and demonstrated that melittin and PLA2 are the main components responsible for this activity type. The outcomes indicated that bee venom components inhibit Gram-negative and Gram-positive bacteria, having an important impact also against *Candida* genus species by inducing antifungal effects [119].

Bee venom has great potential as a wound dressing component, accelerating skin regeneration and promoting the healing process of diabetic wounds, especially due to its anti-inflammatory activity. Hypoxia affects the rate of wound healing by lowering it. Insufficient recruitment of macrophages and neutrophils into the wound environment delays the start of the inflammatory phase and, therefore, the healing process is affected. Hypoxia is also responsible for enhancing the early inflammatory response by prolonging inflammatory cytokines release. The oxidative stress is enhanced by hyperglycemia, leading to various dysfunctions affecting T cell immunity and phagocytosis and causing dysfunctions of fibroblasts. The accumulation of reactive oxygen species (ROS) increases cellular damage, which is also affecting neovascularization. The healing process is also affected by lower angiogenesis, impaired lymphangiogenesis, and a higher rate of apoptosis [120]. This apitherapeutic agent has numerous other clinical applications in autoimmune diseases and osteoarthritis and in neurodegenerative affections, including Parkinson’s and Alzheimer’s. Due to its antinociceptive activity, bee venom is responsible for reducing and treating chronic pain. Amine constituents have an important impact on the nervous system due to their biological effects [121].

## 3. Skin Regeneration Applications

### 3.1. Formulations Based on Honey

Over the years, various studies have been conducted on the use of honey in treating skin injuries due to the antimicrobial and anti-inflammatory properties that this natural product possesses. Hence, honey has often been incorporated into hydrogels to streamline wound healing. This bee product enhances fibroblast and macrophage activities in the wound environment lowers protease function and raises hemoglobin oxygen release. The wound is sterilized by the presence of hydrogen peroxide, which is also responsible for promoting the development of endothelial vascular growth factors [122].

El-kased et al. evaluated the healing potential of hydrogel formulation with different concentrations of incorporated honey (25%, 50%, and 75% *w*/*w*), obtained through the cold mechanical method. Half of the samples used chitosan as a polymer, while the other half used a polyacrylic acid polymer or Carbopol 934. The samples were investigated in vitro to see honey’s release profile using dialysis bags and in vivo on 10 albino mice. The results indicated that the honey release profile depends on the type of polymer used in the hydrogel and on the percentage of honey. The largest amount of honey released is recorded in the case of 75% honey chitosan hydrogel, while the smallest amount is shown in the case where the honey concentration was 25%, and the polymer was Carbopol 934. An explanation could be related to the rheology of Carbopol due to the multiple cross-linkages per polymer. Honey hydrogel samples were tested against the most common strains responsible for bacterial infections: *P. aeruginosa*, *S. aureus*, *Klebsiella pneumonia*, and S. *pyogenes* using the disc diffusion antibiotic sensitivity test. According to the results, as the amount of honey increases, so does the ability to inhibit bacteria, and regarding the type of hydrogel, honey-based chitosan showed better antimicrobial activity than Carbopol 934 [123].

Movaffagh et al. also evaluated the healing potential of honey chitosan hydrogel on skin mice after an induced third-degree burn. Regarding the histopathological indices, the test was performed on Wistar rats, and the examination was made on days 3, 7, and 14. Initially, the proportion of healing was similar for all the samples, but after two weeks of treatment, the highest re-epithelization was identified in the case of pure honey. The histopathological sections confirmed that using hydrogel-based honey and chitosan showed major healing outcomes, indicated by the development of new blood vessels and epidermal regeneration. The researchers demonstrated that the samples with a higher amount of honey are more efficient in the healing process and not only in bacteria inhibition [124].

Combarros-Fuertes et al. increased the area of investigation in another study where they evaluated the antibacterial potential of three different types of honey on the same substrate of bacteria. They investigated avocado honey (AH), chestnut honey (ChH), and polyfloral honey (PH) and their effects on *E. coli* and *S. aureus*. These types of honey were chosen due to their superior antibacterial properties from up to 16 types of honey. The results indicated that the samples with polyfloral and chestnut honey seem to have small improvements compared to those with avocado honey, but the differences are quite small [125].

To improve its antimicrobial activity, Jenkins and Cooper combined honey with antibiotics and tested the resulting formulations against *S. aureus*. Samples containing antibiotics or antibiotics and 5% manuka honey were tested against *Epidemic Methicillin-Resistant S. aureus (EMRSA-15).* According to the results, samples without honey showed a decreased inhibitory activity compared with samples containing honey. The addition of honey in the case of Imipenem and Rifampicin antibiotics indicated a significant improvement in the diameter of inhibition, followed by Tetracycline, Erythromycin, and Mupirocin. In the case of Gentamicin, the addition of honey decreased the inhibition diameter. The addition of honey showed improved inhibition activity against *EMRSA-15* in almost all the antibiotics except Cephalexin and Amoxicillin, where no improvement was observed; antibiotics with or without honey had the same inhibitory activity [126].

Researchers investigated the antibacterial and skin wound healing effects of a hydrogel sheet based on honey, chitosan, and gelatin in another study. According to the outcomes, the samples without honey or chitosan indicated a lower inhibitory activity against *E. coli* and *S. aureus* (50%) than the samples containing honey-loaded chitosan-gelatin hydrogel which indicated 100% effectiveness on these bacteria strains. These results reveal that honey’s addition to chitosan hydrogels enhances the antibacterial effect. Compared with commercial ointment, the hydrogel sheet indicated a 20% higher healing in the burn area [127].

Formulation of curcumin and honey incorporated in chitosan and alginate by simple mixing and using an in situ polymerization method indicated a good swelling capacity and bio adhesion, good drug diffusion, good water vapor transmission, and well as a great tensile strength. The obtained dressings accelerated the re-epithelization process and induced tissue granulation according to the in vivo tests, and in one week, the wound was completely healed [128].

Another interesting work of Rafati et al. refers to the development of bio-nanocomposite hydrogel wound dressings by the freezing–thawing cyclic method. The hydrogel incorporates natural antibiotics such as honey, clay nanoparticles, polyvinyl alcohol (PVA), and egg white [129]. The hydrogel properties such as swelling and dehydration were optimized by the increased number of clay nanoparticles. The honey release was also controlled by the clay nanoparticles. Outcomes of in vivo tests indicated a decrease in the wound surface of all animals, and the bio-nanocomposite hydrogel showed better healing than in the case of the control group. The properties of these bio-nanocomposite enhance the healing process especially due to their ability to maintain a wet environment in the wound area and due to the proteins from the egg white [130].

Santos et al. developed a formulation based on PVA and sodium carboxymethylcellulose, starch, and gelation to prepare manuka honey-based cryogels. Honey cryogels indicated the highest swelling capacities, degradation, and lower gel fractions. According to the results, the manuka honey solutions indicated an inhibitory activity against *S. aureus* due to the nonperoxide components. The concentration of manuka honey determines the sample’s effectiveness and acts like a co-adjuvant to antibiotics when applied against bacteria strains [131]. Other researchers focused on obtaining a hydrogel-based drug delivery system using colloidal nanosilver as a base for honey incorporation to enhance wound healing activity. The results indicated a better healing activity of formula containing 3.5% chitosan and 40% honey along with nanosilver due to chitosan properties, as it accelerates the epithelialization process and, therefore, the rate of wound healing. Honey has a significant role in modulating wound healing when incorporated into chitosan. These parameters assure a cost-effective, nontoxic, natural honey hydrogel delivery system that improves healing, and further investigations could be developed for clinical applications [132].

### 3.2. Formulations Based on Propolis

As in the case of honey, the biological effects of propolis are related to the origin and type of these bee products. The diversity of propolis attracts different properties, and the best-studied propolis is the poplar type. Governa et al. evaluated the antibacterial property of poplar propolis against Gram-positive and Gram-negative strains, including *S. aureus*, *S. epidermidis*, *S. pyogenes*, *Streptococcus pneumoniae* (*S. pneumoniae*), *E. coli*, and *P. aeruginosa.* Poplar propolis was more efficient against Gram-positive bacteria than Gram-negative bacteria, except *S. epidermis* [133]. Comparative with the antimicrobial activity of honey, which is more effective against Gram-negative bacteria, one can state that propolis and honey have different actions regarding antibacterial properties.

Abdullah et al. also investigated the antimicrobial activity of propolis, but they used another type, namely the propolis of the *H. itama* stingless bee species. The results of the study indicated that the inhibition zone of the *S. aureus* and *B. subtilis* were significantly higher than those of *E. coli*, which registered a lower inhibition zone. A small difference was identified in the inhibition zone of *P. aeruginosa*, meaning that propolis particles do not have significant inhibitory activity against this type of bacteria, specifically on Gram-negative strains. When compared with commercial antibiotics, propolis indicated better antibacterial activity, which means that propolis can be used as a natural antibiotic in the fight against infections [134].

In current years, researchers focused on mixing honey and propolis to establish if there is a synergistic effect in the treatment of wounds. Afonso et al. investigated the antioxidant activity of the honey and propolis mix and its in vitro wound healing activity. Dark brown honey showed the highest antioxidant activity, while light yellow indicated low antioxidant by βCarotene bleaching test. In the case of propolis samples, the most concentrated was observed to be the most effective as an antioxidant, while the mixture of honey and propolis registered an improvement towards samples with only honey. The propolis concentration in the mixture showed small differences in the efficiency as an antioxidant; therefore, the antioxidant activity is not related so much to the concentration but to the combination of propolis with honey. Regarding the anti-inflammatory activity, according to the results, the addition of propolis indicated a better anti-inflammatory activity than the samples with honey or propolis extract [135].

In another study, Abbaszadeh et al. investigated the effects of propolis incorporated in a chitosan biofilm in the process of wound healing. The results showed that the group treated with propolis and chitosan nanoparticles biofilm indicated a higher contraction area, significant improvements were observed after day 9, and the best results were obtained after 21 days. Compared with the other groups, the most efficient seems to be the treatment with propolis pasta. In all cases, the wound contraction area decreased progressively, but the most suitable treatment was applied for groups treated with a propolis and chitosan nanoparticles biofilm [136].

A different proposed strategy to enhance the multiple biological properties of propolis was the encapsulation of the bee product into liposomes. Aytekin et al. developed a drug delivery system by encapsulating Turkish propolis into liposomes using a mix of the main phosphatidylcholine stabilized with ascorbyl palmitate named phospholipon 90G as lipid matrix, using an ethanol injection method. They focused on in vitro evaluation of the antioxidant and antimicrobial activity of this system and demonstrated that encapsulated propolis extract indicated a better antimicrobial inhibition than free propolis against Gram-negative and Gram-positive bacteria and also suggested a significant inhibitory impact against fungi such as *C. krusei* and *C. albicans*. After the encapsulation, the DPPH assay indicated that the antioxidant capacity was maintained and pointed out the importance of these nanoparticles’ implementation into wound dressing agents or additives in dermal treatments [137].

Another piece of research on in vivo and in vitro studies has indicated that propolis combined with polymeric wound dressings has an important impact on their usability in wound healing management. In a diabetic wound healing model, the propolis–PVA nanofiber wound dressing showed a faster wound closure after one week of treatment. On male rats with second-degree burns, propolis associated with natural rubber membrane enhances re-epithelization, collagen deposition, and regeneration and induces a decrease in the number of inflammatory cells in the animals [138]. In another study, researchers obtained a bilayer wound dressing based on a polycaprolactone/gelatin scaffold electrospun on a dense membrane. The results showed a reduced wound closure time due to the bilayer dressing and improved biodegradability, biocompatibility, and mechanical proprieties of dressings in wound management. The protection against infections in the wound is exhibited by the propolis component in the first layer of the dressing, which has a strong antibacterial and antifungal activity [139].

### 3.3. Formulations Based on Royal Jelly

Many studies have focused on the antimicrobial activity of royal jelly and the in vitro and in vivo wound healing properties of the antibacterial peptide defensin-1. Nascimento et al. investigated the biological characteristics of Brazilian royal jelly against the following bacteria: *S. aureus*, *E. coli*, *Proteus mirabilis*, *S. epidermidis*, *S. pneumoniae*, *Klebsiella pneumoniae*, *S. enteritidis*, and *P. aeruginosa.* According to the results, the royal jelly was effective against all bacteria. Still, the most susceptible to the samples was *S. pneumoniae*, which means that this bee product can be used in healing wound burns due to the presence of fatty acid 10H2DA [140].

Bucekova et al. continued the investigations into royal jelly and focused especially on defensin-1 antibacterial peptides and evaluated its potential in wound healing management. The obtained results indicated a significant increase in the wound healing rate, related to the higher concentration of water royal jelly. The presence of mitomycin has an influence only on the lower concentrations of water royal jelly extract, where the re-epithelization of the wound area was delayed. Compared with controls, the water royal jelly extract exhibited higher cell migration rates due to the chemoattractant properties of keratinocytes. Regarding the effects of r-Def-1, the wound closure rates exhibited a significant increase. After 24 h, the wound was almost complete after the treatment with r-Def-1. The wounds treated with royal jelly and defensin-1 indicated a more significant wound closure rate than the control, and after day 15, the epithelization was complete. An enhanced re-epithelization was observed on the wound treated with royal jelly and defensin-1, and after day 7, new blood vessels were formed. Therefore, similar wound healing activities were identified in the wound treated with royal jelly and defensin-1 peptide, which indicated that this type of treatment accelerates the healing process [84].

Royal jelly components like the fatty acid 10H2DA and defensin-1 peptide accelerated the wound healing process and made this bee product suitable for developing a wound dressing. The studies that focused on the antimicrobial properties of royal jelly demonstrated significant improvements in the re-epithelization process and the treatment of bacterial infections. According to another study, the inhibitory activity of 10-HDA against Paenibacillus larvae was significantly increased with the decrease of pH. 10-HDA is also highly effective against bacterial pathogens, including *E. coli* (*hemolytic*), *Staphylococcusxylosus*, *S. aureus*, *Staphylococcus intermedius B*, *Streptococcus alactolyticus*, *Salmonella cholearasuis*, and *Vibro parahaemolyticus* [141].

Monica Mierzejewski compared the antimicrobial activity of honey, propolis, and royal jelly to establish their effectiveness and also made a comparison with some common antibiotics to see if these natural products may replace the effects of typical antibiotics. The tests were made against *Staphylococcus aureus*, *E. coli*, *S. epidermidis*, and *Bacillus cereus* (*B. cereus*) [142]. According to the test results, honey exhibited significant inhibitory activity against *S. aureus*, *E. coli*, and *B. cereus* when compared with royal jelly and propolis, and the most reduced zone of inhibition was against *S. epidermis*. Royal jelly was the most effective against *S. epidermidis,* with a value quite similar to Kanamycin. Both propolis and royal jelly indicated similar inhibition values against *B. cereus,* and propolis exhibited the lowest efficiency against *E. coli.* Among all the antibiotics, penicillin indicated the worst inhibitory activity against all bacteria, while Kanamycin was the most efficient antibiotic against all bacteria except *S. aureus*, which was more inhibited by Tetracycline. Comparatively, honey was the most effective sample against *E. coli*, *B. cereus*, and *S. aureus* among all the bee products, followed by royal jelly with a significant impact against *S. epidermidis*. The lowest antimicrobial activity was registered by propolis, especially on *E. coli*. Honey and royal jelly, when compared with antibiotics, showed small differences toward Kanamycin and Tetracycline.

### 3.4. Formulations Based on Bee Pollen

Various studies focused on the important activities of bee pollen, its potential in the medical field, and its pathological features, especially on the fact that bee pollen modulates the burn wound healing process. Kaškonienė et al. compared the most important properties of natural bee pollen with pasteurized polyfloral bee pollen. Some samples were spontaneously fermented, and others used *Lactobacillus rhamnosus* and *Lactococcus lactis* bacteria to complete the fermentation process. The results indicated that the natural microflora of the bee pollen helps the fermentation process and provides a better antimicrobial and antioxidant activity (natural bee pollen) than the pasteurized bee pollen. The bee pollen’s biological activity was enhanced by the fermentation process. The improvements were dependent on the botanical origin of the pollen and also on the fermentation type (spontaneous or with added bacteria) [143].

Spulber et al. investigated the antimicrobial activity of fresh bee pollen collected from different apiaries in Romania and concluded that bee pollen ethanolic extracts possess different antimicrobial properties against various pathogenic species of microorganisms related to the botanical origins of pollen. The most sensitive bacteria is *S. aureus* to rape pollen extract. According to the results, bee pollen extracts exhibited a stronger antimicrobial activity against Gram-positive bacteria than Gram-negative bacteria species [144].

Kacaniova et al. revealed that microbial strains showed variable susceptibility depending on the solvent used to extract the bee pollen sample. *E. coli* has been the most susceptible type of bacteria when tested against a 70% ethanol extract. When compared with a 96% ethanol extract, the sample showed a lower impact on the strains. On *P. aeruginosa*, the most insignificant impact was registered, regardless of the solvent extracts. Therefore, the strain specificity depends on the used solvent (methanol or ethanol) or concentration (96% or 70% ethanol) [145].

### 3.5. Formulations Based on Beeswax

Gümüş and Karaman Özlü planned to investigate the effect of a mixture of beeswax, olive oil, and *A. tinctoria* (L.) *Tausch* on burn wounds to determine the impact on burn healing, pain during dressing changes, and duration of hospital stay. The researchers determined that the dressing applied to the experimental group of patients accelerated the process of epithelialization, reduced hospitalization durations, reduced the levels of pain experienced by the patients during dressing, and completely prevented wound site infections in the experimental group. These results suggest that this mixture may be considered an effective burn dressing for the care of second-degree burns [146].

In another study, Hromiš et al. added caraway essential oil and beeswax to chitosan to design natural, biodegradable, bioactive (antioxidant and antimicrobial) packaging film with good barrier properties towards water vapor, as well as air oxygen. The results suggested that mechanical properties of chitosan film were affected, thickness grew significantly, while the addition of beeswax deteriorated tensile properties and elongation. Moreover, beeswax addition lowered chitosan film sensitivity to ambient humidity, swelling in different pH and water solubility, widening the area of possible application of these films. In addition, when beeswax was added to the chitosan–caraway film, the water vapor transmission rate was significantly lowered [147].

Bayir et al. evaluated beeswax–olive oil–butter mixture and impregnated it into a bandage to be applied to a second-degree burn rat model. The mixture enhanced burn wound healing and contributed to skin renewal by modulating tissue factors such as TGF-β1 and VEGF-α. The outcomes indicated that bandages based on beeswax, olive oil, and butter mixture exhibited an improved therapeutic effect on the second-degree burn area. The results showed that the positive effects of this treatment can be observed in skin regeneration and wound management [107].

Another study made by Moustafa and Atiba compared the healing of deep second-degree burns treated with silver sulfadiazine and a mixture of honey, beeswax, and olive oil. The results indicated a decrease in inflammation and exudation following the mixture treatment more than silver sulfadiazine treatment and control but not significantly. The anti-inflammatory effects of mixture treatment can resolve the inflammatory process induced by burn injury. The mechanism of the therapeutic effects of the mixture on burn lesions might be attributed to the elevation of nitric oxide in the lesions, the inhibition of fungal or bacterial growth, the inhibition of leukotriene B4, and its antioxidant and anti-inflammatory activities [148].

An in vivo study developed by Ebrahimpour et al. indicated an acceleration of the healing process in second-degree burns on rats by using refined calcium hydroxide powder combined with beeswax and sesame oil [149]. According to the outcomes of all these studies, bee products possess specific antimicrobial activities against different types of bacteria and can be used as a potential natural antibiotic in the infection’s treatment.

### 3.6. Formulations Based on Bee Venom

The bactericidal and bacteriostatic activity of bee venom is related to the presence of melittin, which exhibits an important inhibitory effect against *S. pyrogenes*, *S. aureus,* and *S. epidermis*. Moreover, melittin has an impact in viral skin infections and fungi due to its capacity to affect and destroy the cell wall of the bacteria. Bee venom has great potential also against fungi, mostly inhibiting *C. albicans*, *Trichophyton mentagrophytes*, *Malassezia furfur*, and *Trichophyton* [85].

Researchers demonstrated that bee venom has an important impact on wound healing management thanks to its antioxidant, analgesic, anti-inflammatory, and antibacterial features. A topical formulation based on bee venom incorporated in chitosan films was applied to healing diabetic rats’ wounds. The outcomes revealed that the formulation showed biocompatibility to the skin and bee venom enhanced the anti-inflammatory activity of chitosan films. Moreover, the increase in collagen and b-defensin-2 (BD-2) expression indicated a higher rate of wound closure in diabetic mice [150].

In another study, the potential of melittin was investigated against a large spectrum of fungi, and it was demonstrated that micromolar concentrations of this peptide have a potent activity among them. Moreover, according to the results of the study, the mechanisms responsible for preventing fungus from growing were identified, such as modifications in fungal gene expression, permeabilization of the membrane, synthesis inhibition of (1,3)-β-d-glucan, and induction of apoptosis by ROS-mediated mitochondria/caspase-dependent pathway [151].

Another group of researchers focused on obtaining a hydrogel-based PVA and chitosan with bee venom incorporated and observed that the addition of bee venom improved the morphological, physical, and mechanical features. Moreover, the antioxidant and anti-inflammatory activities of the hydrogel enhanced the wound healing process in case of skin regeneration in diabetic patients. Topical preparations seem to minimize the systematic side effects; therefore, hydrogel formulations have good predictability regarding patient compliance [152].

The anti-inflammatory activity of apamin was also investigated on TNF-α and IFN-γ-induced inflammatory responses in human keratinocytes in case of atopic dermatitis. The activation of transcription factors such as JAK/STAT and NF-kB is prevented by the presence of apamin from the bee venom; these factors are correlated with inflammatory cytokines in TNF-α and IFN-β-treated human keratinocytes. According to the results, in atopic dermatitis, apamin plays a significant role especially due to its anti-inflammatory functions [153].

## 4. Remarks and Future Perspectives

Bee-derived products exhibit significant inhibitory activities against bacteria and can be used to treat wounds. Several studies suggested that bee products can replace the antimicrobial activity and efficiency of antibiotics, but further investigation is needed to establish the potential of a topical mixture including honey, royal jelly, and propolis.

The efficiency of honey against Gram-negative bacteria was better than in the case of Gram-positive bacteria, and the regeneration properties were significantly improved due to the special properties possessed by honey. Propolis indicated a significant inhibitory activity against Gram-positive bacteria and remarkable antioxidant activity, meaning that its addition can enhance the biological properties of honey. Royal jelly instead exhibited antimicrobial activity against specific bacteria types for which honey and royal jelly indicated low inhibitory activity. The fatty acid and the defensin-1 peptide in the composition of royal jelly showed an improvement in wound healing management. The application of bee pollen has good effects on local pain elimination and prevention of platelet aggregation, while the antibacterial and antifungal compounds of beeswax affect the production of cytokines by skin cells. These bee products seem to complete each other’s deficiencies, and their combination may have a better impact on the wound healing process.

Interestingly, a reduced number of studies investigated the activity of all these products mixed, and they focused on the properties of each product, not on their mixture. Together, these products may have more enhanced properties than their separate entities; therefore, next steps should be taken in this direction.

## Figures and Tables

**Table 2:** The pharmacological activity of chemical propolis compounds—adapted from [58].

Chemical Compounds	Pharmacological Activity
Acacetin	Anti-inflammatory
Apigenin	Anti-inflammatory; Antimicrobial
Artepilin C	Antitumor activity; Antioxidative
Caffeic acid phenethyl ester	Antitumor activity; Anti-inflammatory
Chrysin	Anti-inflammatory; Antibacterial
Caffeic acid	Antifungal; Antiviral; Anti-inflammatory
Cinnamic acid	Anti-inflammatory
Dicaffeoylquinic acid derivatives	Hepatoprotective
Ferulic acid, Galangin, Gallic acid	Anti-inflammatory
Protocatechuic acid	Anti-inflamatory; Antibacterial
Pinocembrin	Antifungal; Antimold; Local anesthesia
Propofol	Antioxidative

**Table 3:** Bee pollen—the biological properties and main mechanisms (Reprinted from [98]).

Property	Description of the Biological Activity’s Mechanism
Nutritive properties	Presence of carbohydrates, proteins, lipids, exogenous amino acids, unsaturated fatty acids, phytosterols, bioelements, phospholipids, and vitamins.
Antioxidative properties	Complexing metals; hydroxyl radicals are eliminated.
Cardioprotective properties	Inhibition of blood platelets aggregation and ACE activity are inhibited.
Hepatoprotective properties	The activity of detoxifying in industrial poisoning; lipofuscin reduction.
Anti-inflammatory properties	Inhibitory activity of NO production and COX-2.
Antibacterial properties	Bacteria metabolism is affected in the case of *Pseudomonas aeruginosa* (*P. aeruginosa*), *Staphylococcus epidermis* (*S. epidermis*), *B. cerus*, *E. coli*, *S. aureus*, *Listeria monocytogenes* (*L. monocytogenes*), *B. subtilis*, and *Salmonella enterica*
Anticarcinogenic properties	*Brassica camperstris* L. bee pollen extract is responsible for decreasing the expression of antiapoptotic proteins and for caspase-3 enzyme activity increase; 17β-estradiol activity is inhibited by *Salix alba* L. and *Cistus incanus* L. bee pollens.
Antianaemic properties	Hemoglobin level is increasing while blood platelets number is decreasing.
Bone tissue effects	*Cystus ladaniferus* L. bee pollen is responsible for increasing alkaline phosphatase level, resorption inhibition of the femur, and osteoclastic cell formation.

## Data Availability

Not applicable.

## References

[1] Ozhathil D.K., Wolf S.E. (2022). Prevention and treatment of burn wound infections: The role of topical antimicrobials. Expert Rev. Anti-Infect. Ther..

[2] Pavel T.I., Chircov C., Rădulescu M., Grumezescu A.M. (2020). Regenerative wound dressings for skin cancer. Cancers.

[3] Tottoli E.M., Dorati R., Genta I., Chiesa E., Pisani S., Conti B. (2020). Skin Wound Healing Process and New Emerging Technologies for Skin Wound Care and Regeneration. Pharmaceutics.

[4] Warby R., Maani C.V. (2021). Burn Classification.

[5] Stoica A.E., Chircov C., Grumezescu A.M. (2020). Hydrogel Dressings for the Treatment of Burn Wounds: An Up-To-Date Overview. Materials.

[6] Niculescu A.G., Grumezescu A.M. (2022). An Up-to-Date Review of Biomaterials Application in Wound Management. Polymers.

[7] Xu J., Su M., Jin Z., Zhou W., Sun Y., Jin Y., Shi Z. (2022). Effects of Natural Brown Cotton Bleached Gauze on Wound Healing. Materials.

[8] Oba J., Okabe M., Yoshida T., Soko C., Fathy M., Amano K., Kobashi D., Wakasugi M., Okudera H. (2020). Hyperdry human amniotic membrane application as a wound dressing for a full-thickness skin excision after a third-degree burn injury. Burn. Trauma.

[9] Barski D., Gerullis H., Ecke T., Varga G., Boros M., Pintelon I., Timmermans J.-P., Otto T. (2018). Human amniotic membrane dressing for the treatment of an infected wound due to an entero-cutaneous fistula: Case report. Int. J. Surg. Case Rep..

[10] Cui R., Zhang L., Ou R., Xu Y., Xu L., Zhan X.-Y., Li D. (2022). Polysaccharide-Based Hydrogels for Wound Dressing: Design Considerations and Clinical Applications. Front. Bioeng. Biotechnol..

[11] Debele T.A., Su W.-P. (2022). Polysaccharide and protein-based functional wound dressing materials and applications. Int. J. Polym. Mater. Polym. Biomater..

[12] Rivero G., Meuter M., Pepe A., Guevara M.G., Boccaccini A.R., Abraham G.A. (2020). Nanofibrous membranes as smart wound dressings that release antibiotics when an injury is infected. Colloids Surf. A Physicochem. Eng. Asp..

[13] Schulte-Werning L.V., Murugaiah A., Singh B., Johannessen M., Engstad R.E., Škalko-Basnet N., Holsæter A.M. (2021). Multifunctional Nanofibrous Dressing with Antimicrobial and Anti-Inflammatory Properties Prepared by Needle-Free Electrospinning. Pharmaceutics.

[14] Hussain Z., Thu H.E., Rawas-Qalaji M., Naseem M., Khan S., Sohail M. (2022). Recent developments and advanced strategies for promoting burn wound healing. J. Drug Deliv. Sci. Technol..

[15] Shalaby M.A., Anwar M.M., Saeed H. (2022). Nanomaterials for application in wound Healing: Current state-of-the-art and future perspectives. J. Polym. Res..

[16] Neacsu I.A., Melente A.E., Holban A.M., Ficai A., Ditu L.M., Kamerzan C.M., Tihauan B.M., Nicoara A.I., Bezirtzoglou E., Chifiriuc M.C. (2019). Novel hydrogels based on collagen and ZnO nanoparticles with antibacterial activity for improved wound dressings. Rom. Biotechnol. Lett..

[17] Radulescu M., Ficai D., Oprea O., Ficai A., Andronescu E., Holban A.M. (2015). Antimicrobial Chitosan based Formulations with Impact on Different Biomedical Applications. Curr. Pharm. Biotechnol..

[18] Neacsu I.A., Leau S.A., Marin S., Holban A.M., Vasile B.S., Nicoara A.I., Ene V.L., Bleotu C., Kaya M.G.A., Ficai A. (2021). Collagen-Carboxymethylcellulose Biocomposite Wound-Dressings with Antimicrobial Activity. Materials.

[19] Paduraru A., Ghitulica C., Trusca R., Surdu V.A., Neacsu I.A., Holban A.M., Birca A.C., Iordache F., Vasile B.S. (2019). Antimicrobial Wound Dressings as Potential Materials for Skin Tissue Regeneration. Materials.

[20] Radulescu D.-M., Neacsu I.A., Grumezescu A.-M., Andronescu E. (2022). New Insights of Scaffolds Based on Hydrogels in Tissue Engineering. Polymers.

[21] Williams D.F., Atala A., Lanza R., Mikos A.G., Nerem R. (2019). Chapter 36—Hydrogels in Regenerative Medicine. Principles of Regenerative Medicine.

[22] Negut I., Grumezescu V., Grumezescu A.M. (2018). Treatment Strategies for Infected Wounds. Molecules.

[23] Luo X., Dong Y., Gu C., Zhang X., Ma H. (2021). Processing Technologies for Bee Products: An Overview of Recent Developments and Perspectives. Front. Nutr..

[24] Mello B.C.B.S., Petrus J.C.C., Hubinger M.D. (2010). Concentration of flavonoids and phenolic compounds in aqueous and ethanolic propolis extracts through nanofiltration. J. Food Eng..

[25] Pellati F., Prencipe F.P., Bertelli D., Benvenuti S. (2013). An efficient chemical analysis of phenolic acids and flavonoids in raw propolis by microwave-assisted extraction combined with high-performance liquid chromatography using the fused-core technology. J. Pharm. Biomed. Anal..

[26] Ciulu M., Spano N., Pilo M.I., Sanna G. (2016). Recent Advances in the Analysis of Phenolic Compounds in Unifloral Honeys. Molecules.

[27] Pyrzynska K., Biesaga M. (2009). Analysis of phenolic acids and flavonoids in honey. TrAC Trends Anal. Chem..

[28] López-Gutiérrez N., Aguilera-Luiz M.d.M., Romero-González R., Vidal J.L.M., Garrido Frenich A. (2014). Fast analysis of polyphenols in royal jelly products using automated TurboFlow™-liquid chromatography–Orbitrap high resolution mass spectrometry. J. Chromatogr. B.

[29] Sawicki T., Starowicz M., Kłębukowska L., Hanus P. (2022). The Profile of Polyphenolic Compounds, Contents of Total Phenolics and Flavonoids, and Antioxidant and Antimicrobial Properties of Bee Products. Molecules.

[30] Minden-Birkenmaier B.A., Bowlin G.L. (2018). Honey-Based Templates in Wound Healing and Tissue Engineering. Bioengineering.

[31] Meo S.A., Al-Asiri S.A., Mahesar A.L., Ansari M.J. (2017). Role of honey in modern medicine. Saudi J. Biol. Sci..

[32] Albaridi N.A. (2019). Antibacterial Potency of Honey. Int. J. Microbiol..

[33] Ramsay E.I., Rao S., Madathil L., Hegde S.K., Baliga-Rao M.P., George T., Baliga M.S. (2019). Honey in oral health and care: A mini review. J. Oral Biosci..

[34] Cianciosi D., Forbes-Hernandez T.Y., Afrin S., Gasparrini M., Reboredo-Rodriguez P., Manna P.P., Zhang J., Bravo Lamas L., Martinez Florez S., Agudo Toyos P. (2018). Phenolic Compounds in Honey and Their Associated Health Benefits: A Review. Molecules.

[35] Nguyen H.T.L., Panyoyai N., Kasapis S., Pang E., Mantri N. (2019). Honey and Its Role in Relieving Multiple Facets of Atherosclerosis. Nutrients.

[36] Habryka C., Socha R., Juszczak L. (2020). The Effect of Enriching Honey with Propolis on the Antioxidant Activity, Sensory Characteristics, and Quality Parameters. Molecules.

[37] Al-Ghamdi A.A., Ansari M.J. (2021). Biological and therapeutic roles of Saudi Arabian honey: A comparative review. J. King Saud Univ.-Sci..

[38] Nolan V.C., Harrison J., Cox J.A.G. (2019). Dissecting the Antimicrobial Composition of Honey. Antibiotics.

[39] Almasaudi S. (2021). The antibacterial activities of honey. Saudi J. Biol. Sci..

[40] Martinotti S., Ranzato E. (2018). Honey, Wound Repair and Regenerative Medicine. J. Funct. Biomater..

[41] Iftikhar A., Nausheen R., Mukhtar I., Iqbal R.K., Raza A., Yasin A., Anwar H. (2022). The regenerative potential of honey: A comprehensive literature review. J. Apic. Res..

[42] Khan S.U., Anjum S.I., Rahman K., Ansari M.J., Khan W.U., Kamal S., Khattak B., Muhammad A., Khan H.U. (2018). Honey: Single food stuff comprises many drugs. Saudi J. Biol. Sci..

[43] Da Silva B., Caon T., Mohr E.T.B., Biluca F.C., Gonzaga L.V., Fett R., Dalmarco E.M., Costa A.C.O. (2022). Phenolic profile and in vitro anti-inflammatory activity of Mimosa scabrella Bentham honeydew honey in RAW 264.7 murine macrophages. J. Food Biochem..

[44] Osés S.M., Cantero L., Puertas G., Fernández-Muiño M.Á., Sancho M.T. (2022). Antioxidant, antimicrobial and anti-inflammatory activities of ling-heather honey powder obtained by different methods with several carriers. LWT.

[45] De-Melo A., Almeida-Muradian L., Sancho M., Pascual Maté A. (2017). Composition and properties of Apis mellifera honey: A review. J. Apic. Res..

[46] Vică M.L., Glevitzky M., Tit D.M., Behl T., Heghedűş-Mîndru R.C., Zaha D.C., Ursu F., Popa M., Glevitzky I., Bungău S. (2021). The antimicrobial activity of honey and propolis extracts from the central region of Romania. Food Biosci..

[47] Kavanagh S., Gunnoo J., Marques Passos T., Stout J.C., White B. (2019). Physicochemical properties and phenolic content of honey from different floral origins and from rural versus urban landscapes. Food Chem..

[48] Ahmed S., Sulaiman S.A., Baig A.A., Ibrahim M., Liaqat S., Fatima S., Jabeen S., Shamim N., Othman N.H. (2018). Honey as a Potential Natural Antioxidant Medicine: An Insight into Its Molecular Mechanisms of Action. Oxid. Med. Cell. Longev..

[49] Zabaiou N., Fouache A., Trousson A., Baron S., Zellagui A., Lahouel M., Lobaccaro J.A. (2017). Biological properties of propolis extracts: Something new from an ancient product. Chem. Phys. Lipids.

[50] Oryan A., Alemzadeh E., Moshiri A. (2018). Potential role of propolis in wound healing: Biological properties and therapeutic activities. Biomed. Pharm..

[51] Alvear M., Santos E., Cabezas F., Pérez-Sanmartín A., Lespinasse M., Veloz J. (2021). Geographic Area of Collection Determines the Chemical Composition and Antimicrobial Potential of Three Extracts of Chilean Propolis. Plants.

[52] Przybyłek I., Karpiński T.M. (2019). Antibacterial Properties of Propolis. Molecules.

[53] Soós Á., Bódi É., Várallyay S., Molnár S., Kovács B. (2022). Element composition of propolis tinctures prepared from Hungarian raw propolis. LWT.

[54] Nalbantsoy A., Sarıkahya N.B., Özverel C.S., Barlas A.B., Kırcı D., Akgün İ.H., Yalçın T., Düven G., Kışla D., Demirci B. (2022). Chemical composition and biological activities of Cypriot propolis. J. Apic. Res..

[55] Mutlu C., Özer-Atakoğlu Ö., Erbaş M., Yalçın M.G. (2022). Advances in the Elemental Composition Analysis of Propolis Samples from Different Regions of Turkey by X-ray Fluorescence Spectrometry. Biol. Trace Elem. Res..

[56] Rufatto L.C., dos Santos D.A., Marinho F., Henriques J.A.P., Roesch Ely M., Moura S. (2017). Red propolis: Chemical composition and pharmacological activity. Asian Pac. J. Trop. Biomed..

[57] Alsayed M.F.S., Hashem A., Al-Hazzani A.A., Abd_Allah E.F. (2020). Biological control of yeast contamination of industrial foods by propolis. Saudi J. Biol. Sci..

[58] Toreti V.C., Sato H.H., Pastore G.M., Park Y.K. (2013). Recent progress of propolis for its biological and chemical compositions and its botanical origin. Evid.-Based Complementary Altern. Med. Ecam..

[59] Galeotti F., Maccari F., Fachini A., Volpi N. (2018). Chemical Composition and Antioxidant Activity of Propolis Prepared in Different Forms and in Different Solvents Useful for Finished Products. Foods.

[60] Šuran J., Cepanec I., Mašek T., Radić B., Radić S., Tlak Gajger I., Vlainić J. (2021). Propolis Extract and Its Bioactive Compounds—From Traditional to Modern Extraction Technologies. Molecules.

[61] Martinotti S., Ranzato E. (2015). Propolis: A new frontier for wound healing?. Burn. Trauma.

[62] Sforcin J.M. (2016). Biological Properties and Therapeutic Applications of Propolis. Phytother. Res..

[63] Anjum S.I., Ullah A., Khan K.A., Attaullah M., Khan H., Ali H., Bashir M.A., Tahir M., Ansari M.J., Ghramh H.A. (2019). Composition and functional properties of propolis (bee glue): A review. Saudi J. Biol. Sci..

[64] Freitas A.S., Cunha A., Oliveira R., Almeida-Aguiar C. (2022). Propolis antibacterial and antioxidant synergisms with gentamicin and honey. J. Appl. Microbiol..

[65] Aljadaan S.A.N., Elias R.S., Al-Anssari R.A. (2020). Investigation of the Antioxidant and Antibacterial Activity of Novel Quercetin Derivatives. Biointerface Res. Appl. Chem..

[66] Selamoglu Z., Sevindik M., Bal C., Ozaltun B., Sen I., Pasdaran A. (2020). Antioxidant, antimicrobial and DNA protection activities of phenolic content of Tricholoma virgatum (Fr.) P.Kumm. Biointerface Res. Appl. Chem..

[67] Uslu M.E., Mele A., Bayraktar O. (2019). Evaluation of the hemostatic activity of Equisetum arvense extract: The role of varying phenolic composition and antioxidant activity due to different extraction conditions. Biointerface Res. Appl. Chem..

[68] Cao X.-P., Chen Y.-F., Zhang J.-L., You M.-M., Wang K., Hu F.-L. (2017). Mechanisms underlying the wound healing potential of propolis based on its in vitro antioxidant activity. Phytomedicine.

[69] Pasupuleti V.R., Sammugam L., Ramesh N., Gan S.H. (2017). Honey, Propolis, and Royal Jelly: A Comprehensive Review of Their Biological Actions and Health Benefits. Oxid. Med. Cell. Longev..

[70] Braakhuis A. (2019). Evidence on the Health Benefits of Supplemental Propolis. Nutrients.

[71] Franchin M., Freires I.A., Lazarini J.G., Nani B.D., da Cunha M.G., Colón D.F., de Alencar S.M., Rosalen P.L. (2018). The use of Brazilian propolis for discovery and development of novel anti-inflammatory drugs. Eur. J. Med. Chem..

[72] Hassan A.A.-m., Elenany Y.E., Nassrallah A., Cheng W., Abd El-Maksoud A.A. (2022). Royal jelly improves the physicochemical properties and biological activities of fermented milk with enhanced probiotic viability. LWT.

[73] Mullen A. (2021). Ancient residues, dietary clues. Nat. Food.

[74] Ahmad S., Campos M.G., Fratini F., Altaye S.Z., Li J. (2020). New Insights into the Biological and Pharmaceutical Properties of Royal Jelly. Int. J. Mol. Sci..

[75] Ramanathan A., Nair A., Sugunan V. (2018). A review on Royal Jelly proteins and peptides. J. Funct. Foods.

[76] Fratini F., Cilia G., Mancini S., Felicioli A. (2016). Royal Jelly: An ancient remedy with remarkable antibacterial properties. Microbiol. Res..

[77] Kocot J., Kielczykowska M., Luchowska-Kocot D., Kurzepa J., Musik I. (2018). Antioxidant Potential of Propolis, Bee Pollen, and Royal Jelly: Possible Medical Application. Oxid. Med. Cell. Longev..

[78] Kim B.Y., Lee K.S., Jung B., Choi Y.S., Kim H.K., Yoon H.J., Gui Z.-Z., Lee J., Jin B.R. (2019). Honeybee (Apis cerana) major royal jelly protein 4 exhibits antimicrobial activity. J. Asia-Pac. Entomol..

[79] Maleki V., Jafari-Vayghan H., Saleh-Ghadimi S., Adibian M., Kheirouri S., Alizadeh M. (2019). Effects of Royal jelly on metabolic variables in diabetes mellitus: A systematic review. Complementary Ther. Med..

[80] Khazaei M., Ansarian A., Ghanbari E. (2018). New Findings on Biological Actions and Clinical Applications of Royal Jelly: A Review. J. Diet. Suppl..

[81] Alreshoodi F., Sultanbawa Y. (2015). Antimicrobial Activity of Royal Jelly. Anti-Infect. Agents.

[82] Cornara L., Biagi M., Xiao J., Burlando B. (2017). Therapeutic Properties of Bioactive Compounds from Different Honeybee Products. Front. Pharmacol..

[83] Lin Y., Shao Q., Zhang M., Lu C., Fleming J., Su S. (2019). Royal jelly-derived proteins enhance proliferation and migration of human epidermal keratinocytes in an in vitro scratch wound model. BMC Complementary Altern. Med..

[84] Bucekova M., Sojka M., Valachova I., Martinotti S., Ranzato E., Szep Z., Majtan V., Klaudiny J., Majtan J. (2017). Bee-derived antibacterial peptide, defensin-1, promotes wound re-epithelialisation in vitro and in vivo. Sci. Rep..

[85] Kurek-Górecka A., Górecki M., Rzepecka-Stojko A., Balwierz R., Stojko J. (2020). Bee Products in Dermatology and Skin Care. Molecules.

[86] Maghsoudlou A., Sadeghi A., Mohebodini H., Toldrá F. (2019). Royal Jelly: Chemistry, Storage and Bioactivities. J. Apic. Sci..

[87] Maqsoudlou A., Mahoonak A.S., Mora L., Mohebodini H., Toldrá F., Ghorbani M. (2019). Peptide identification in alcalase hydrolysated pollen and comparison of its bioactivity with royal jelly. Food Res. Int. Ott. Ont..

[88] Ghosh S., Jung C. (2020). Changes in nutritional composition from bee pollen to pollen patty used in bumblebee rearing. J. Asia-Pac. Entomol..

[89] Mosić M., Trifković J., Vovk I., Gašić U., Tešić Ž., Šikoparija B., Milojković-Opsenica D. (2019). Phenolic Composition Influences the Health-Promoting Potential of Bee-Pollen. Biomolecules.

[90] Karabagias I., Karabagias V., Gatzias I., Riganakos K. (2018). Bio-Functional Properties of Bee Pollen: The Case of “Bee Pollen Yoghurt”. Coatings.

[91] Adaškevičiūtė V., Kaškonienė V., Kaškonas P., Barčauskaitė K., Maruška A. (2019). Comparison of Physicochemical Properties of Bee Pollen with Other Bee Products. Biomolecules.

[92] Khalifa S.A.M., Elashal M.H., Yosri N., Du M., Musharraf S.G., Nahar L., Sarker S.D., Guo Z., Cao W., Zou X. (2021). Bee Pollen: Current Status and Therapeutic Potential. Nutrients.

[93] Duan H., Dong Z., Li H., Li W.R., Shi S.X., Wang Q., Cao W.G., Fang X.M., Fang A.D., Zhai K.F. (2019). Quality evaluation of bee pollens by chromatographic fingerprint and simultaneous determination of its major bioactive components. Food Chem. Toxicol. Int. J. Publ. Br. Ind. Biol. Res. Assoc..

[94] Castagna A., Benelli G., Conte G., Sgherri C., Signorini F., Nicolella C., Ranieri A., Canale A. (2020). Drying Techniques and Storage: Do They Affect the Nutritional Value of Bee-Collected Pollen?. Molecules.

[95] Thakur M., Nanda V. (2020). Composition and functionality of bee pollen: A review. Trends Food Sci. Technol..

[96] Kieliszek M., Piwowarek K., Kot A., Błażejak S., Chlebowska-Śmigiel A., Wolska I. (2018). Pollen and bee bread as new health-oriented products: A review. Trends Food Sci. Technol..

[97] Sforcin J., Bankova V., Kuropatnicki A. (2017). Medical Benefits of Honeybee Products. Evid.-Based Complementary Altern. Med..

[98] Rzepecka-Stojko A., Stojko J., Kurek-Górecka A., Górecki M., Kabała-Dzik A., Kubina R., Moździerz A., Buszman E. (2015). Polyphenols from Bee Pollen: Structure, Absorption, Metabolism and Biological Activity. Molecules.

[99] Didaras N.A., Karatasou K., Dimitriou T.G., Amoutzias G.D., Mossialos D. (2020). Antimicrobial Activity of Bee-Collected Pollen and Beebread: State of the Art and Future Perspectives. Antibiotics.

[100] Mărgăoan R., Stranț M., Varadi A., Topal E., Yücel B., Cornea-Cipcigan M., Campos M.G., Vodnar D.C. (2019). Bee Collected Pollen and Bee Bread: Bioactive Constituents and Health Benefits. Antioxidants.

[101] Denisow B., Denisow-Pietrzyk M. (2016). Biological and therapeutic properties of bee pollen: A review. J. Sci. Food Agric..

[102] Khan S., Lari Q.H., Khan M. (2016). Therapeutic Uses of Mom Zard (Beeswax) in Unani System of Medicine—A Review. J. Anal. Pharm. Res..

[103] Lima W.G., Brito J.C.M., da Cruz Nizer W.S. (2020). Bee products as a source of promising therapeutic and chemoprophylaxis strategies against COVID-19 (SARS-CoV-2). Phytother. Res. PTR.

[104] Navarro-Hortal M.D., Orantes-Bermejo F.J., Sánchez-González C., Varela-López A., Giampieri F., Torres Fernández-Piñar C., Serra-Bonvehí J., Forbes-Hernández T.Y., Reboredo-Rodríguez P., Llopis J. (2019). Industrial-Scale Decontamination Procedure Effects on the Content of Acaricides, Heavy Metals and Antioxidant Capacity of Beeswax. Molecules.

[105] Amin M., Putra N., Kosasih E.A., Prawiro E., Luanto R.A., Mahlia T.M.I. (2017). Thermal properties of beeswax/graphene phase change material as energy storage for building applications. Appl. Therm. Eng..

[106] Szulc J., Machnowski W., Kowalska S., Jachowicz A., Ruman T., Steglińska A., Gutarowska B. (2020). Beeswax-Modified Textiles: Method of Preparation and Assessment of Antimicrobial Properties. Polymers.

[107] Bayir Y., Un H., Ugan R., Akpinar E., Calik I., Halici Z. (2019). The effects of Beeswax, Olive oil and Butter impregnated bandage on burn wound healing. Burns.

[108] Bogdanov S. (2016). Beeswax: Production, Properties, Composition, Control. Beeswax Book.

[109] Karabey T., KaragÖZoĞLu Ş. (2019). Using Beeswax, Olive Oil and Centaury Oil for Pressure Ulcers. Turk. Klin. J. Case Rep..

[110] Zhang Y., Simpson B.K., Dumont M.-J. (2018). Effect of beeswax and carnauba wax addition on properties of gelatin films: A comparative study. Food Biosci..

[111] Fratini F., Cilia G., Turchi B., Felicioli A. (2016). Beeswax: A minireview of its antimicrobial activity and its application in medicine. Asian Pac. J. Trop. Med..

[112] Nader R.A., Mackieh R., Wehbe R., El Obeid D., Sabatier J.M., Fajloun Z. (2021). Beehive Products as Antibacterial Agents: A Review. Antibiotics.

[113] Sakka A.E., Abdulrhman M., Shehata I.H. (2013). Comparison between topical application of Honey, Bees wax and Olive Oil Propolis extract and Nystatin for treatment of Diaper Dermatitis in Infants. Int. J. Pediatrics Child Health.

[114] Kim D.-H., Lee H.-W., Park H.-W., Lee H.-W., Chun K.-H. (2020). Bee venom inhibits the proliferation and migration of cervical-cancer cells in an HPV E6/E7-dependent manner. BMB Rep..

[115] Sİg A.K., Güney M., Özlem Ö.Z., Hüseyin Ş.A.N. (2019). Bee venom: A medical perspective. Turk. J. Clin. Lab..

[116] Wehbe R., Frangieh J., Rima M., El Obeid D., Sabatier J.-M., Fajloun Z. (2019). Bee Venom: Overview of Main Compounds and Bioactivities for Therapeutic Interests. Molecules.

[117] Lin T.-Y., Hsieh C.-L. (2020). Clinical Applications of Bee Venom Acupoint Injection. Toxins.

[118] Jang S., Kim K.H. (2020). Clinical Effectiveness and Adverse Events of Bee Venom Therapy: A Systematic Review of Randomized Controlled Trials. Toxins.

[119] Carpena M., Nuñez-Estevez B., Soria-Lopez A., Simal-Gandara J. (2020). Bee Venom: An Updating Review of Its Bioactive Molecules and Its Health Applications. Nutrients.

[120] Kurek-Górecka A., Komosinska-Vassev K., Rzepecka-Stojko A., Olczyk P. (2021). Bee Venom in Wound Healing. Molecules.

[121] Weis W.A., Ripari N., Conte F.L., Honorio M.d.S., Sartori A.A., Matucci R.H., Sforcin J.M. (2022). An overview about apitherapy and its clinical applications. Phytomedicine Plus.

[122] El-Ashram S., El-Samad L.M., Basha A.A., El Wakil A. (2021). Naturally-derived targeted therapy for wound healing: Beyond classical strategies. Pharmacol. Res..

[123] El-Kased R.F., Amer R.I., Attia D., Elmazar M.M. (2017). Honey-based hydrogel: In vitro and comparative In vivo evaluation for burn wound healing. Sci. Rep..

[124] Movaffagh J., Fazly Bazzaz B.S., Yazdi A.T., Sajadi-Tabassi A., Azizzadeh M., Najafi E., Amiri N., Taghanaki H.B., Ebrahimzadeh M.H., Moradi A. (2019). Wound Healing and Antimicrobial Effects of Chitosan-hydrogel/Honey Compounds in a Rat Full-thickness Wound Model. Wounds A Compend. Clin. Res. Pract..

[125] Combarros-Fuertes P., Estevinho L.M., Teixeira-Santos R., Rodrigues A.G., Pina-Vaz C., Fresno J.M., Tornadijo M.E. (2020). Antibacterial Action Mechanisms of Honey: Physiological Effects of Avocado, Chestnut, and Polyfloral Honey upon Staphylococcus aureus and Escherichia coli. Molecules.

[126] Jenkins R., Cooper R. (2012). Improving antibiotic activity against wound pathogens with manuka honey in vitro. PLoS ONE.

[127] Prasathkumar M., Sadhasivam S. (2021). Chitosan/Hyaluronic acid/Alginate and an assorted polymers loaded with honey, plant, and marine compounds for progressive wound healing—Know-how. Int. J. Biol. Macromol..

[128] Liu H., Wang C., Li C., Qin Y., Wang Z., Yang F., Li Z., Wang J. (2018). A functional chitosan-based hydrogel as a wound dressing and drug delivery system in the treatment of wound healing. RSC Adv..

[129] Rafati Z., Sirousazar M., Hassan Z.M., Kheiri F. (2020). Honey-Loaded Egg White/Poly(vinyl alcohol)/Clay Bionanocomposite Hydrogel Wound Dressings: In Vitro and In Vivo Evaluations. J. Polym. Environ..

[130] Nezhad-Mokhtari P., Javanbakht S., Asadi N., Ghorbani M., Milani M., Hanifehpour Y., Gholizadeh P., Akbarzadeh A. (2021). Recent advances in honey-based hydrogels for wound healing applications: Towards natural therapeutics. J. Drug Deliv. Sci. Technol..

[131] Santos A., Moreira A., Piler Carvalho C., Luchese R., Prudencio E., McGuinness G., Mendes M., Oliveira R. (2019). Physically Cross-Linked Gels of PVA with Natural Polymers as Matrices for Manuka Honey Release in Wound-Care Applications. Materials.

[132] Abraham S.A., Yashavanth G., Deveswaran R., Bharath S., Azamathulla M., Shanmuganathan S. (2022). Honey based hydrogel as delivery system for wound healing. Mater. Today Proc..

[133] Governa P., Cusi M.G., Borgonetti V., Sforcin J.M., Terrosi C., Baini G., Miraldi E., Biagi M. (2019). Beyond the Biological Effect of a Chemically Characterized Poplar Propolis: Antibacterial and Antiviral Activity and Comparison with Flurbiprofen in Cytokines Release by LPS-Stimulated Human Mononuclear Cells. Biomedicines.

[134] Abdullah N.A., Ja’afar F., Yasin H.M., Taha H., Petalcorin M.I.R., Mamit M.H., Kusrini E., Usman A. (2019). Physicochemical analyses, antioxidant, antibacterial, and toxicity of propolis particles produced by stingless bee Heterotrigona itama found in Brunei Darussalam. Heliyon.

[135] Afonso A., Gonçalves J., Luís Â., Gallardo E., Duarte A. (2020). Evaluation of the In Vitro Wound-Healing Activity and Phytochemical Characterization of Propolis and Honey. Appl. Sci..

[136] Abbaszadeh A., Rajabzadeh A., Zarei L. (2019). Effect of Chitosan/Propolis Biodegradable Film on Full-Thickness Wound Healing in Rats. Iran. J. Vet. Surg..

[137] Mendez-Pfeiffer P., Juarez J., Hernandez J., Taboada P., Virués C., Valencia D., Velazquez C. (2021). Nanocarriers as drug delivery systems for propolis: A therapeutic approach. J. Drug Deliv. Sci. Technol..

[138] El-Seedi H.R., Eid N., Abd El-Wahed A.A., Rateb M.E., Afifi H.S., Algethami A.F., Zhao C., Al Naggar Y., Alsharif S.M., Tahir H.E. (2022). Honey Bee Products: Preclinical and Clinical Studies of Their Anti-inflammatory and Immunomodulatory Properties. Front. Nutr..

[139] Eskandarinia A., Kefayat A., Agheb M., Rafienia M., Amini Baghbadorani M., Navid S., Ebrahimpour K., Khodabakhshi D., Ghahremani F. (2020). A Novel Bilayer Wound Dressing Composed of a Dense Polyurethane/Propolis Membrane and a Biodegradable Polycaprolactone/Gelatin Nanofibrous Scaffold. Sci. Rep..

[140] Nascimento A.P., Moraes L.A.R., Ferreira N.U., Moreno G.d.P., Uahib F.G.M., Barizon E.A., Berretta A.A. (2015). The Lyophilization Process Maintains the Chemical and Biological Characteristics of Royal Jelly. Evid.-Based Complementary Altern. Med. Ecam.

[141] Guo J., Wang Z., Chen Y., Cao J., Tian W., Ma B., Dong Y. (2021). Active components and biological functions of royal jelly. J. Funct. Foods.

[142] Mierzejewski M. (2014). The antimicrobial effects of royal jelly, propolis and honey against bacteria of clinical significance in comparison to three antibiotics. Coll. Arts Sci. Biol..

[143] Kaškonienė V., Adaškevičiūtė V., Kaškonas P., Mickienė R., Maruška A. (2020). Antimicrobial and antioxidant activities of natural and fermented bee pollen. Food Biosci..

[144] Spulber R., Doğaroğlu M., Băbeanu N., Popa O. (2018). Physicochemical characteristics of fresh bee pollen from different botanical origins. Rom. Biotechnol. Lett..

[145] Kacaniova M., Vuković N., Chlebo R., Haščík P., Rovná K., Cubon J., Dzugan M., Pasternakiewicz A. (2012). The antimicrobial activity of honey, bee pollen loads and beeswax from Slovakia. Arch. Biol. Sci..

[146] Gümüş K., Karaman Özlü Z. (2017). The Effect of a Beeswax, Olive Oil and *Alkanna tinctoria* (L.) Tausch Mixture on Burn Injuries: An Experimental Study with a Control Group. Complementary Ther. Med..

[147] Hromiš N., Lazić V., Sinisa M., Vaštag Ž., Popović S., Suput D., Džinić N., Velićanski A., Popović L. (2015). Optimization of chitosan biofilm properties by addition of caraway essential oil and beeswax. J. Food Eng..

[148] Moustafa A., Atiba A. (2015). The Effectiveness of a Mixture of Honey, Beeswax and Olive Oil in Treatment of Canine Deep Second-Degree Burn. Glob. Vet..

[149] Ebrahimpour N., Mehrabani M., Iranpour M., Kordestani Z., Mehrabani M., Nematollahi M.H., Asadipour A., Raeiszadeh M., Mehrbani M. (2020). The efficacy of a traditional medicine preparation on second-degree burn wounds in rats. J. Ethnopharmacol..

[150] El-Wahed A.A.A., Khalifa S.A.M., Elashal M.H., Musharraf S.G., Saeed A., Khatib A., Tahir H.E., Zou X., Naggar Y.A., Mehmood A. (2021). Cosmetic Applications of Bee Venom. Toxins.

[151] Memariani H., Memariani M. (2020). Anti-fungal properties and mechanisms of melittin. Appl. Microbiol. Biotechnol..

[152] Chen X., Wang Z., Gao S., Zhang W., Gong H., Xu K., Luo C., Zhi W., Weng J., Li J. (2021). Polyvinyl Alcohol/Chitosan Composite Hydrogels with Sustained Release of Traditional Tibetan Medicine for Promoting Chronic Diabetic Wound Healing. Biomater. Sci..

[153] Gu H., Han S.M., Park K.-K. (2020). Therapeutic Effects of Apamin as a Bee Venom Component for Non-Neoplastic Disease. Toxins.

